# An overview of metal-free synthetic routes to isoxazoles: the privileged scaffold

**DOI:** 10.1039/d1ra04624a

**Published:** 2021-10-06

**Authors:** Soumyadip Das, Kaushik Chanda

**Affiliations:** Department of Chemistry, School of Advanced Sciences, Vellore Institute of Technology Vellore-632014 India chandakaushik1@gmail.com

## Abstract

In the field of drug discovery, isoxazole is a five-membered heterocyclic moiety commonly found in many commercially available drugs. In view of their enormous significance, it is always imperative to unleash new eco-friendly synthetic strategies. Among various novel synthetic techniques in use for isoxazole synthesis, most synthetic methods employ Cu(i) or Ru(ii) as catalysts for (3 + 2) cycloaddition reaction. The particular disadvantages associated with metal-catalyzed reactions are high costs, low abundance, toxicity, a significant generation of waste, and difficulty to separate from the reaction mixtures. In view of these drawbacks, it is always imperative to develop alternate metal-free synthetic routes. This review article highlights a comprehensive overview on the potential application of metal-free synthetic routes for the synthesis of isoxazoles with significant biological interests.

## Abstract 2

Isoxazole, a five-membered heterocyclic moiety commonly found in many commercially available drugs. This review article presents a comprehensive overview on metal-free synthetic routes for the synthesis of isoxazoles with significant bioactivities.

## Graphical abstract

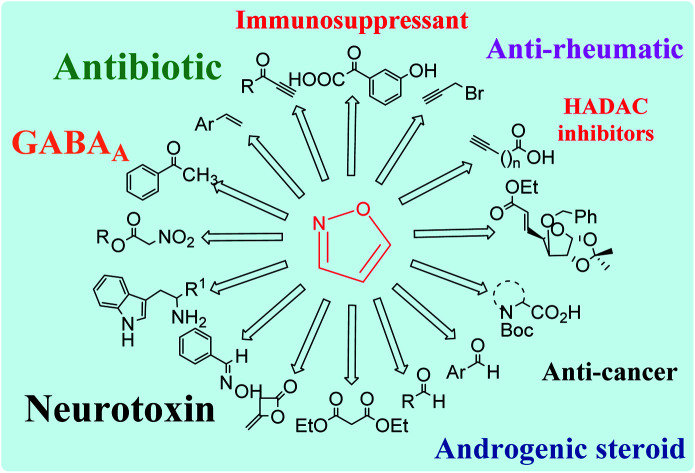

## Introduction

**Fig. 1:**
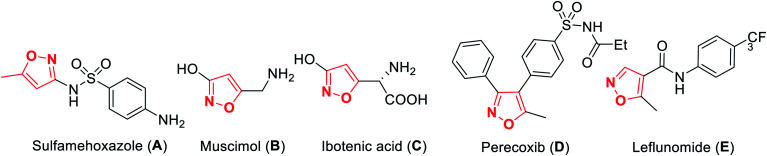
Biologically-active isoxazole-based drug molecules.

A wide variety of functionalized heterocyclic scaffolds and their synthesis are significant to medicinal chemists as they provide the ability to expand the available drug-like chemical space, which bind to the biological targets based on their chemical diversity.^1*a*,*b*^ Furthermore, it is highly desirable to develop robust synthetic methods for the generation of a diverse collection of heterocyclic molecules to accelerate the drug discovery programme. Isoxazole, a five-membered heterocyclic pharmacophore is widely used as a crucial moiety in drug discovery research.^1*c*^ Functionalized isoxazole scaffolds show different biological activities such as anticancer, as potential HDAC inhibitors, antioxidant, antibacterial, and antimicrobial activity.^2^ Evidently, the core structure of isoxazole has been found in many drugs such as sulfamethoxazole^3*a*^A that acts as an antibiotic, muscimol^3*b*^B that acts as GABA_A_, ibotenic acid^3*c*^C that acts as a neurotoxin, parecoxib^3*d*^D that acts as a COX2 inhibitor, and leflunomide^3*e*^E that acts as an immunosuppressant agent (Fig. 1). Moreover, isoxazole derivatives are an important part of small chemical entities, which exists in synthetic goods of day-to-day use.

**Fig. 2:**
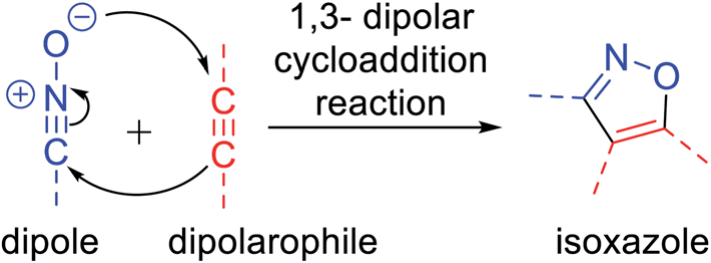
Mechanism of 1,3-dipolar cycloaddition reaction.

Isoxazoles can be synthesized *via* different pathways using both homogeneous as well as heterogeneous catalysts. Nevertheless, the most broadly researched and reported synthesis of isoxazole derivative is through the (3 + 2) cycloaddition reaction of an alkyne that acts as a dipolarophile and nitrile oxide as the dipole.^4^ Two predicted mechanisms have been reported for the 1,3-dipolar cycloaddition reaction—firstly, pericyclic cycloaddition reaction *via* concerted mechanism^5*a*^ and secondly, *via* a step-by-step mechanism through diradical intermediate formation.^5*b*^ Subsequently, the first proposed idea has been accepted, *i.e.*, concerted pathway, *via* the reaction of the dipole and the dipolarophile (Fig. 2). In 2001, Sharpless and his co-workers described this kind of cycloaddition reaction as ‘Click Chemistry’ for the regioselective synthesis of disubstituted triazoles.^6^ In 2005, Fokin *et al.* demonstrated the synthesis of azoles *via* copper(i) catalysis and the DFT study was performed to predict the extraordinary reactivity.^7^ Subsequently, in 2008, Müller *et al.* reported the Pd catalyzed Sonogashira coupling of acid chlorides with terminal alkynes, followed by the 1,3-dipolar cycloaddition with *in situ* prepared nitrile oxides, which resulted in isoxazoles in good yields *via* a one-pot three-component reaction.^8^ Later on, Fokin and his group developed the ruthenium-catalyzed synthetic strategy for the synthesis of 3,4-disubstituted isoxazoles *via* the (3 + 2) cycloaddition reaction.^9^ In 2010, DeKorver *et al.* reported various 1,3-dipolar cycloaddition reactions using copper(ii) catalysts such as copper acetate and copper sulfate pentahydrate.^10^ In the same year, Evano and his group described the copper(ii)-catalyzed 1,3-dipolar cycloaddition reaction using ynamides as a new building block for the synthesis of isoxazoles.^11^ In 2013, we studied the facet-dependent catalytic potential of Cu_2_O nanocrystals as heterogeneous catalysts for the synthesis of isoxazoles.^12^ In the year 2015, Szostak and his co-workers reported different synthetic strategies for the synthesis of substituted isoxazoles using metal catalysts.^13^ In 2018, Nakamura and his group also reported the recent progresses in the synthesis of functionalized isoxazoles *via* 1,3-dipolar cycloaddition, condensation, cycloisomerization, and direct functionalization reactions.^14^ Very recently, Li and his group reported the synthetic sequence leading to isoxazoles *via* the metal-catalyzed cyclization/functionalization of alkynes.^15^ Subsequently, our group demonstrated the microwave-assisted green synthetic pathway for the synthesis of 3,5-disubstituted isoxazoles using a Cu(i) catalyst.^16^

It is well known that metal catalysts and organometallic reagents are widely used for academic research as well as industrial purposes. The metal-mediated synthesis of heterocyclic moieties increases day by day. From the physiological point of view, metals can be divided into important classes, which are required compulsorily in the body for some purpose, and the other ones are non-essential, which are absent in organisms. The second class is different from the previous class as metals that execute toxic effects. However, it has been accepted that essential as well as non-essential metals are simultaneously dangerous, if received in excess.^17^ Homogeneous catalysts are often transition metal complexes and organometallic complexes with high efficiency as well as high selectivity under any reaction environment. However, in reality, the separation of such a metal catalyst from the reaction mixture is very difficult with the generation of waste. Hence, heterogeneous catalysts are generally measured to be technically beneficial over homogeneous catalysts.^18^ However, a considerable interest has been generated for the synthesis of heterocyclic moieties in the metal-free pathway. The synthetic methodology proceeds very well at room temperature conditions with a good range of products in moderate to excellent yields having excellent chemo- and regioselectivity. Further, the metal-free synthetic reactions avoid the requirement of inert gas, cryogenics, as well as toxic metal contamination, making them useful for the generation of diverse molecular architectures.

In continuation of our efforts to explore the potential application of synthesized bioactive compounds in our laboratory,^19^ herein, we report metal-free synthetic routes to isoxazoles and challenges to the future design of isoxazoles as the drug molecule in this review. The synthetic protocols reported in this review provide detailed information of the protocols initiated from the metal-free solid phase and green synthetic processes. Furthermore, assembling all synthetic manipulations on one platform will provide key solutions to resolve the current synthetic problems and also promote knowledge about the current synthetic progress in the synthesis of isoxazole analogs. Subsequently, it may be possible to develop new concepts and increase the diversity of synthetic routes to isoxazole moieties.

## Metal-free solid phase synthesis of isoxazoles

In 2010, Leonetti and his co-workers developed five-membered heterocyclic ligands having a potential binding feature for a therapeutically interesting enzyme. The final aim was to find new compounds having excellent inhibitory activity toward PKTs or other enzymes such as COXs. All the molecules were synthesized through the solid support using Rink amide resin. Initially, polymer-bound 3-hydroxybenzamide 3 was synthesized with the reaction of Rink amide resin 1 and 3-hydroxybenzoic acid 2 using EDC·HCl as the coupling agent in the presence of dry DMF as the solvent under room temperature condition for 12 h. 91% yield was obtained for intermediate 3.

**Scheme 1:**
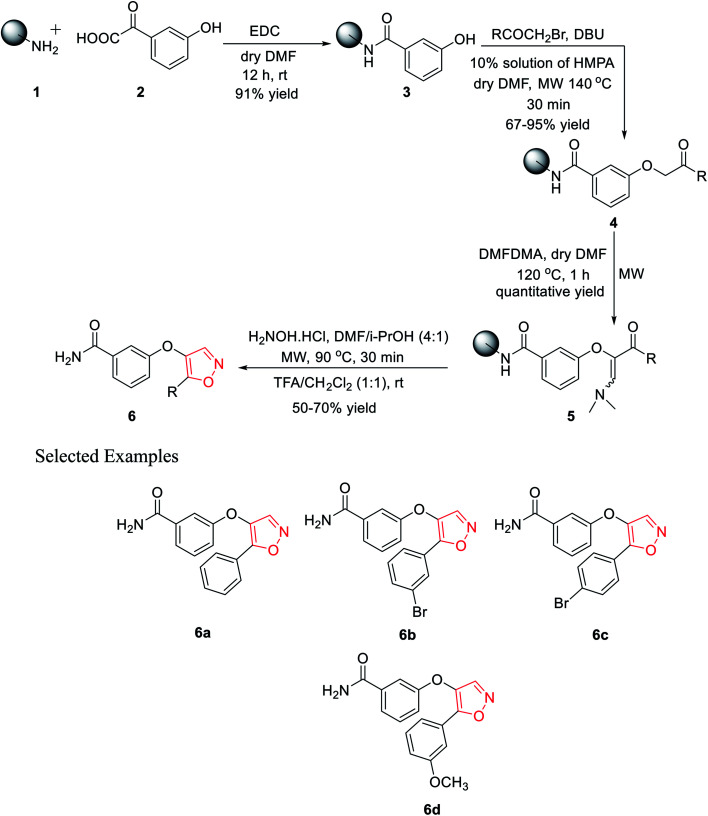
Microwave-assisted solid phase synthesis of 4,5-disubstituted isoxazoles.

In the coupling step, the use of HOBt and DICI as the coupling agents produced a low yield of 78%. Next, different bromomethyl ketones were reacted with solid-supported 3-hydroxybenzamide 3 using 10% mixture of HMPA in DMF with DIPEA as the base under microwave irradiation for excellent yields of the polymer-bound intermediate 4. However, the use of the base DBU in this step reduced the reaction yield to 67%. The next step involved the synthesis of the polymer-bound enaminoketones 5 by the reaction of polymer conjugates 4 with DMFDMA. For the best result, dry DMF was used as the solvent under microwave irradiation at 120 °C for 1 h, leading to quantitative yields. In the final step, five-membered heterocyclization was done by reacting intermediate 5 with hydroxylamine hydrochloride using a solvent mixture (DMF/i-PrOH, 4 : 1) under microwaves at 90 °C for 30 min, followed by cleavage. Finally, the five-membered isoxazoles 6 were obtained in 50–70% yield (Scheme 1).^20^

In the year 2012, Nefzi and his group introduced the diversity-oriented synthesis of isoxazole derivatives by the 1,3-dipolar cycloaddition reaction of alkyne and *in situ* developed nitrile oxides.^21^ The necessary alkyne precursors were developed from the different derivatives of resin-supported carboxylic acids. The solid phase synthesis of all the molecules was done using Houghten's tea-bag approach as the resin was supported with a closed polypropylene mesh.

**Fig. 3:**
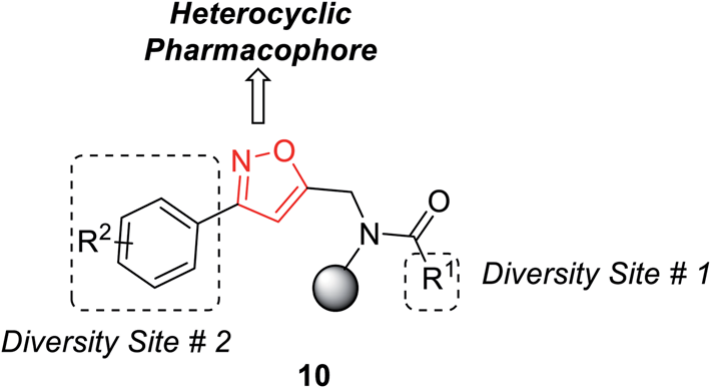
Diversity points of substituted isoxazoles.

The first diversity was presented using different carboxylic acid derivatives (examples: cyclopentanecarboxylic acid, benzoic acid, 1-phenyl-1-cyclopentanecarboxylic acid, 4-biphenylcarboxylic acid, 2-nitrobenzoic acid, piperonylic acid, 1-napthalenecarboxylic, diphenylacetic acid, syringyl, 1-phenyl-1-cyclopropylcarboxylic acid, and 1-cyclopenteneacetic acid) and second diversity was introduced using different *N*-hydroxybenzimidoyl chlorides 9 (Fig. 3).

**Scheme 2:**
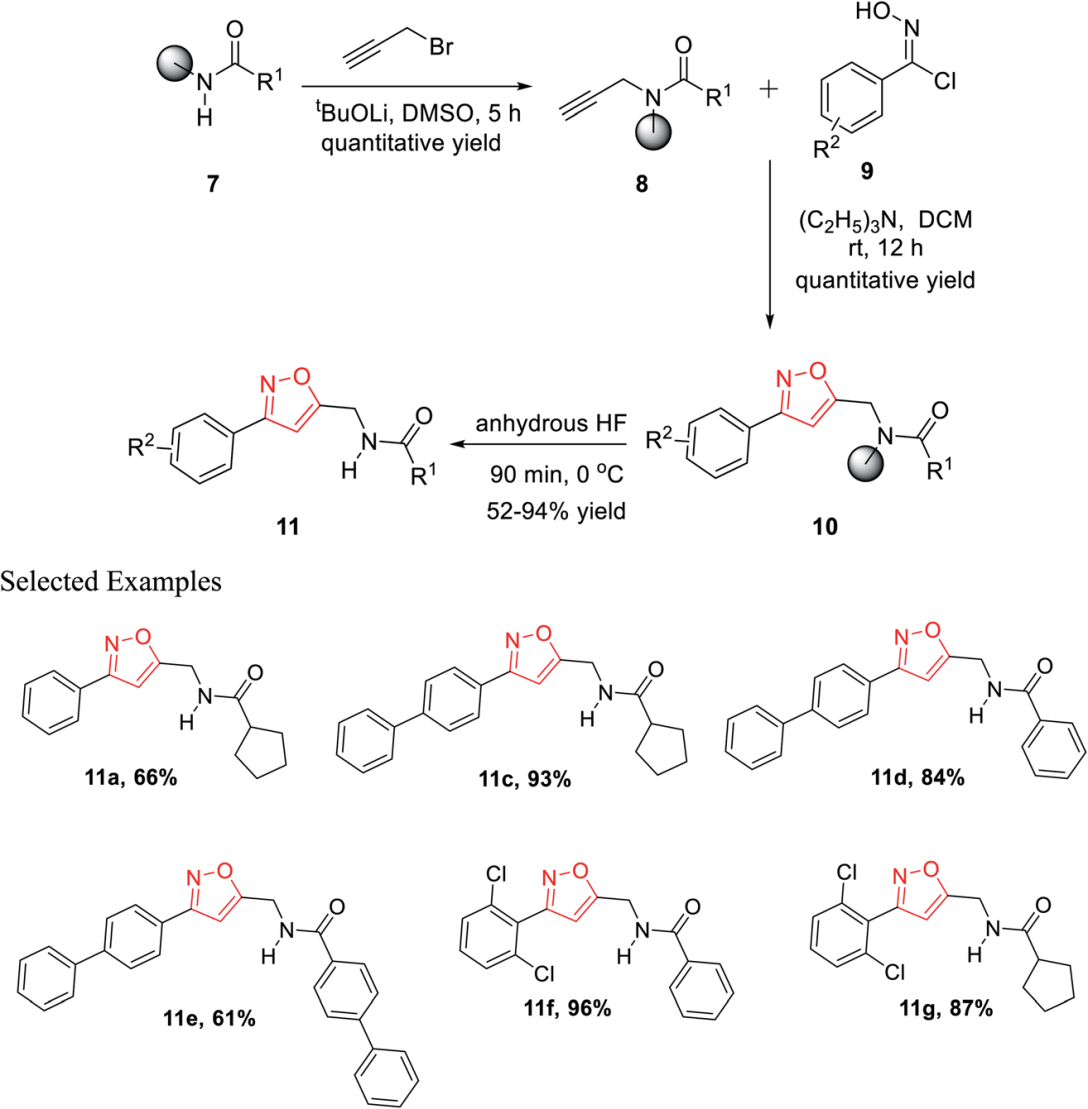
Solid-supported formation of functionalized isoxazoles.

Resin-supported carboxylic acid was reacted with propargyl bromide for 5 h and the produced solid-supported secondary amide 8 in the presence of lithium *t*-butoxide and DMSO as the solvent. Resin-bound isoxazoles 10 were synthesized by the cycloaddition reaction of resin-bound alkynes 8 and *in situ* generated nitrile oxide of the corresponding *N*-hydroxybenzimidoyl chloride derivatives 9. Exceptionally, for resin-supported piperonylic acid, original product along with a diol as the side product were obtained. Finally, the cleavage of the resin from resin-bound isoxazoles was achieved in anhydrous HF for 90 min at 0 °C and finally the targeted product 11 was obtained (Scheme 2).^21^

## Microwave-assisted metal-free routes to substituted isoxazoles

In 2010, Micheli *et al.* reported the synthesis of isoxazole derivatives by the 1,3-dipolar cycloaddition reaction. For a successful 1,3-dipolar cycloaddition reaction, one 1,3-dipole and one dipolarophile is required; in this case, 14 and 15 act as the 1,3-dipole and ethyl-2-chloro-2-(hydroxyimino)acetate acts as the dipolarophile.

**Scheme 3:**
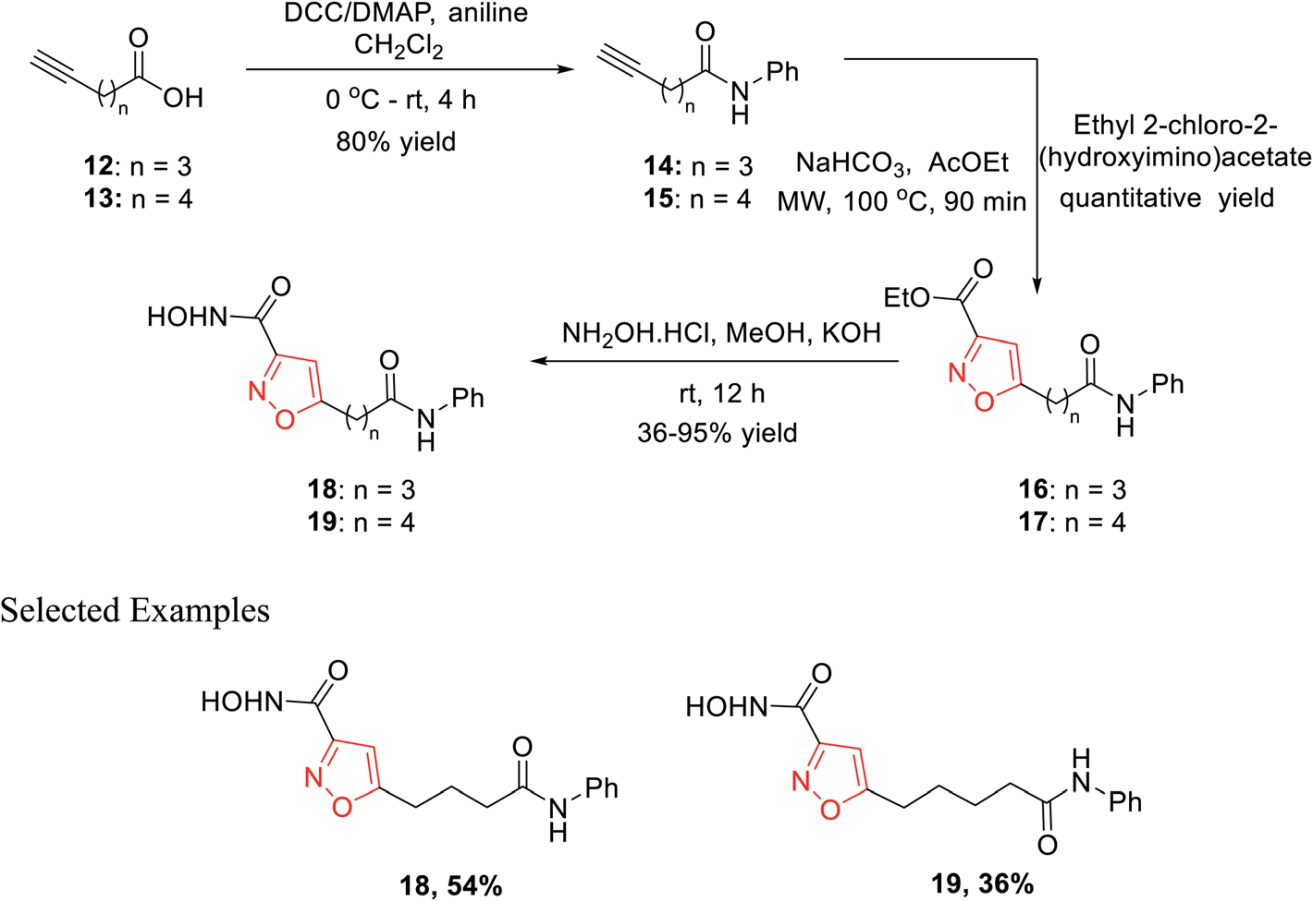
Microwave-assisted metal-free routes to 3,5-disubstituted isoxazoles.

The initial precursor alkynes 14 and 15 were synthesized by the reaction of carboxylic acids 12 and 13 with aniline under conventional coupling conditions using DCC and DMAP as the coupling agents. The *in situ* formed ethoxycarbonyl formonitrile oxide from ethyl-2-chloro-2-(hydroxyimino)acetate was reacted with dipolarophiles 14 and 15 under microwave condition to obtain ester-functionalized isoxazoles 16 and 17 in quantitative yields. Finally, the ester-functionalized isoxazoles were converted hydroxylamine functionalized isoxazole derivatives 18 and 19 by reacting with hydroxylamine hydrochloride in methanolic KOH solution (Scheme 3). All the synthesized derivatives underwent biological fruition to recognize the molecule furnished with histone deacetylase (HDAC) inhibitory action. HDAC inhibitors are a new class of potential anticancer agents, which play a crucial role in epigenetic or non-epigenetic regulation, apoptosis, cell death, and cell cycle arrest in cancer cells. Compound 19 with an isoxazole skeleton has been elected to have inhibitory activity at various HDAC isoforms. The particular compound exhibited 10–70 folds lower IC_50_s at HDAC-6 than HDAC 10, 3, 2, and 1.^22^

In year 2014, Chondrogianni and co-workers developed a fresh pathway for the synthesis of 3,5-disubstituted isoxazole *via* microwave irradiation. Initially, aryl aldehydes 20 were reacted with hydroxylamine hydrochloride and furnished the corresponding oximes 21. Subsequently, oxime derivatives 21 were reacted with TsN(Cl)Na·3H_2_O in the presence of *tert*-butyl alcohol at room temperature, which yielded the most important nitrile oxide intermediate.

**Scheme 4:**
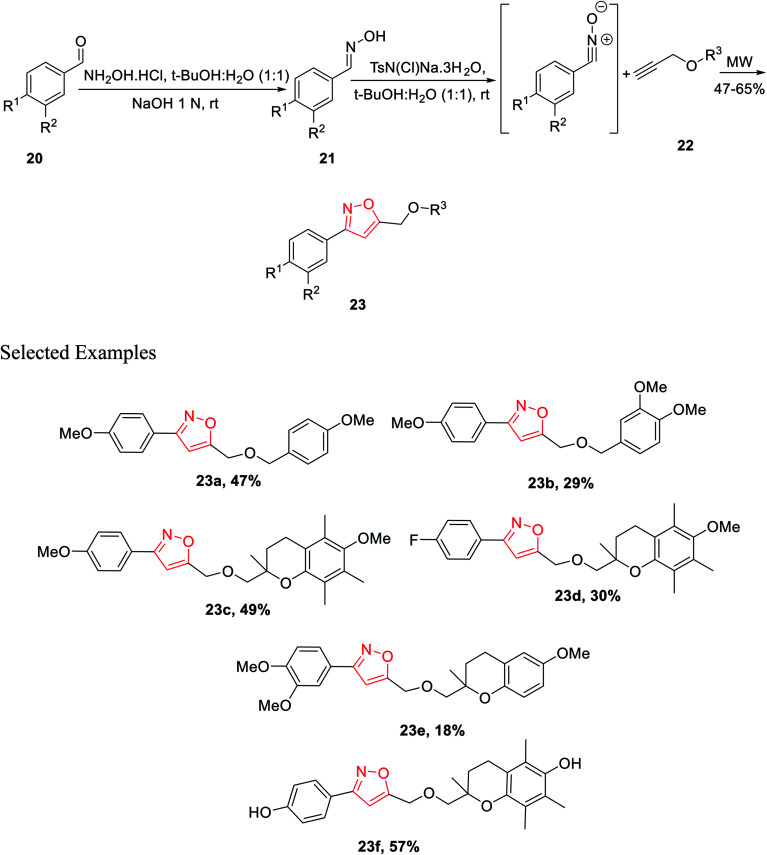
Microwave-assisted synthesis of 3,5-disubstituted isoxazoles.

In the presence of microwave irradiation, functionalized alkynes 22 were reacted with nitrile oxides, which afforded the corresponding substituted isoxazoles 23, while no copper catalysts were used in the reaction medium (Scheme 4). Isoxazole derivatives were tested for the anti-ageing and/or antioxidant properties. The test was performed by following two models—one is the nematode *C. elegans* (*in vivo* model) and other one is human primary fibroblasts (*in vitro* model). Among all the derivatives, compound 23f displayed excellent antioxidant properties compared to the typical antioxidant molecules, for example, quercetin.^23^

**Scheme 5:**
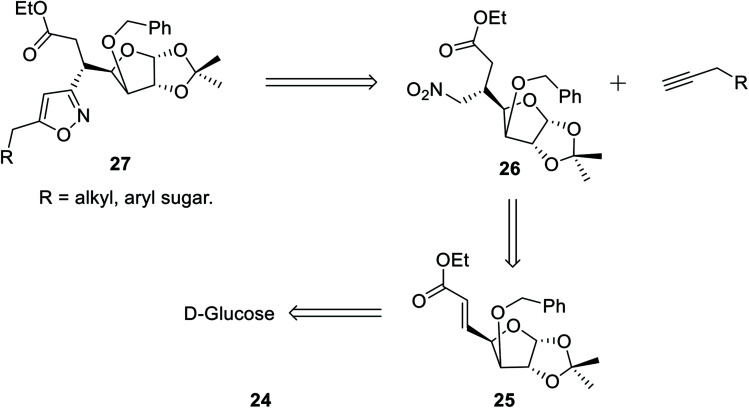
Retrosynthetic analysis for the synthesis of isoxazole-linked glyco-conjugates 27.

In the subsequent year, Tiwari *et al.* reported an extremely regioselective superficial synthesis of unique 3,5-disubstituted isoxazole-linked glyco-conjugates 26 by exploiting the (3 + 2) cycloaddition reaction. The procedure benefited with a prompt and modest way for the initiation of the isoxazole ring at various places of the sugar derivatives with seeming ease. This is a capable method that has been introduced to synthesize the isoxazole-linked glyco-conjugate in the area of carbohydrate chemistry. To synthesize the desired molecule isoxazole-linked glycoconjugate 26, a retrosynthetic study was performed (Scheme 5). d-Glucose 24 was converted to the corresponding glycosyl-β-olefinic ester 25*via* a series of reactions, followed by the reaction with nitromethane to obtain intermediate 26, which underwent the (3 + 2) cycloaddition reaction with alkynes to obtain the target derivatives 27.

**Scheme 6:**
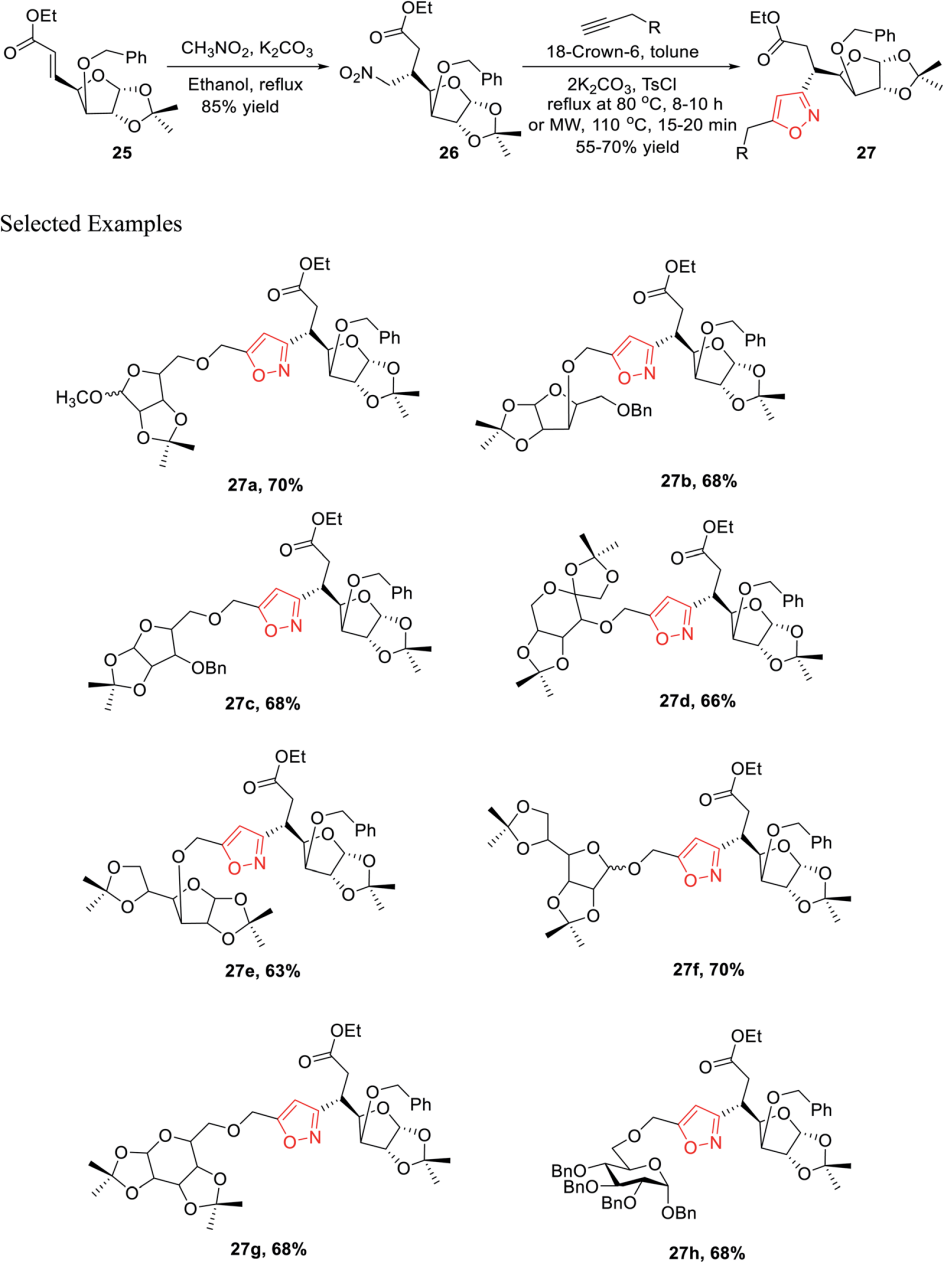
Microwave-assisted regioselective synthesis of unique isoxazole-linked glyco-conjugates.

The synthetic procedure was initiated with inexpensive and easily affordable d-glucose 24, which subsequently underwent a sequence of reactions, such as the protection of isopropylidene, protection of 3-OH using benzyl group, deprotection of 5,6-isopropylidene, oxidation by NaIO_4_, and lastly, olefination *via* the HEW-Wittig method, which yielded glycosyl olefinic ester (1*R*,2*R*,3*S*,4*R*)-ethyl-[3-*O*-benzyl-5,6-dideoxy-1,2-*O*-isopropylidene-d-gluco]-heptfuran-5 enuronate 25.^24*a*,*b*^ Compound 25 was refluxed with nitromethane in anhydrous ethanolic medium with K_2_CO_3_ as the base for 6 h, which yielded (1*R*,2*R*,3*S*,4*R*,5*R*)-ethyl-[3-*O*-benzyl-5,6-dideoxy-1,2-*O*-isopropylidene-5-nitromethyl]-*b*-l-idoheptofuranurnate 26 (Scheme 6). Compound 26 was reacted with alkynes in the presence of 18-crown-6 catalyst, K_2_CO_3_, and 4-toluenesulfonyl chloride at 80 °C for 8–10 h resulted the isoxazole-linked glyco-conjugates 27. However, the application of microwave irradiation reduced the reaction time to 15–20 min at 110 °C to obtain isoxazole-linked glycol-conjugates 27 in good yield. The diversity was introduced in compounds 27 by varying the R groups, *i.e.*, varying different sugars, such as monosaccharides like d-mannose, d-galactose, d-glucose, d-fructose, and d-ribose.^24*c*^

## Metal-free synthesis of isoxazoles *via* miscellaneous methods

**Scheme 7:**
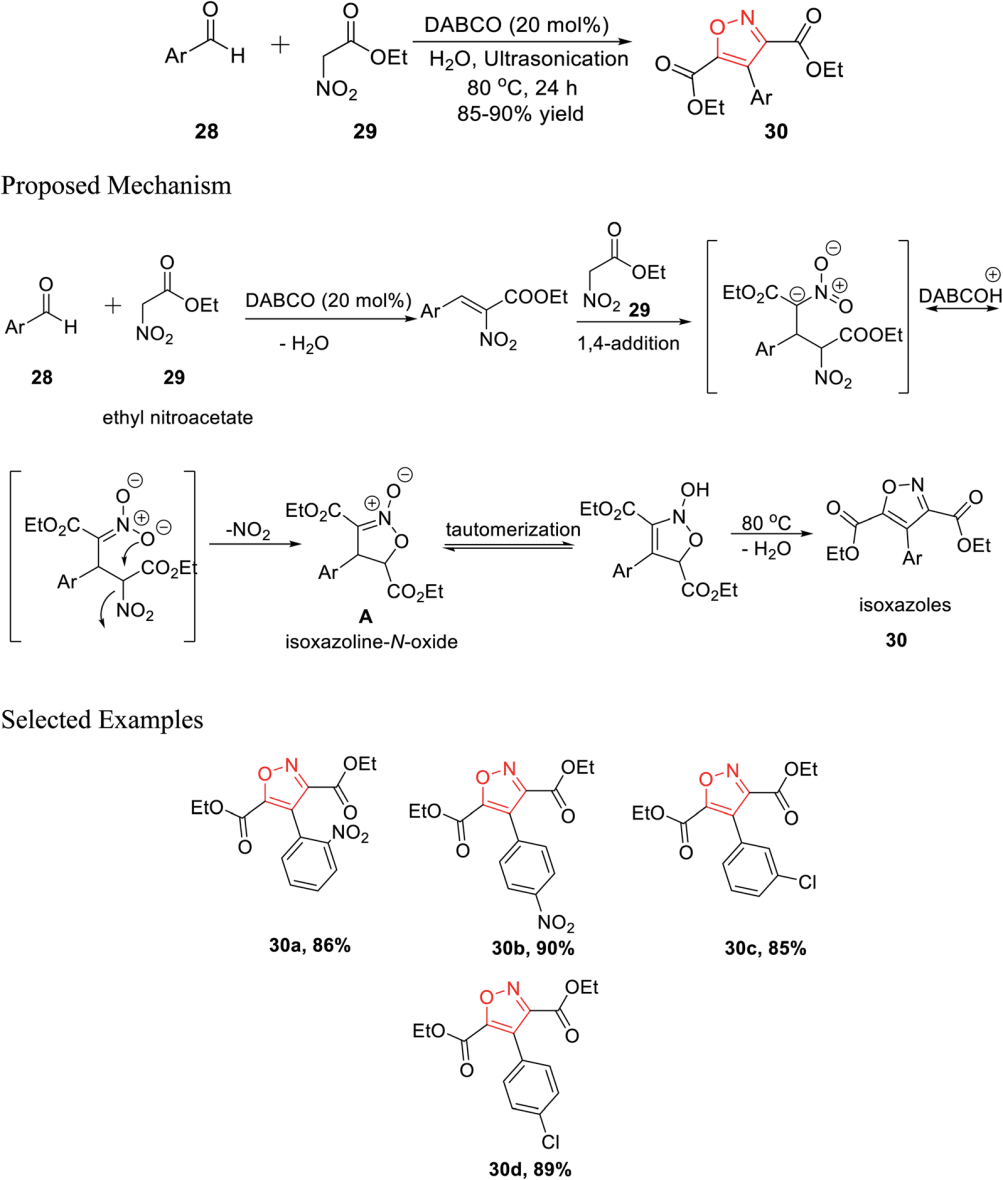
Synthesis of poly substituted isoxazoles *via* a one-pot cascade reaction.

In 2016, Tanyeli's group accomplished a novel method for the formation of isoxazole derivatives by a one-pot cascade reaction *via* the ultrasonication method.^25^ To synthesize the isoxazole derivatives 30, ethyl nitroacetate 29 and aromatic aldehyde 28 were reacted in water using 20 mol% DABCO as the catalyst at 80 °C for 24 h under ultrasonication (Scheme 7).

Different kinds of solvent were used such as toluene, hexane, methanol, ethanol, DCM, chloroform, DMF, DMSO, and, THF but the best result was obtained using water as the solvent. Mechanistically, during the reaction, the corresponding isoxazoline *N*-oxide intermediate A was isolated, which leads to the final product isoxazoles 30*via* tautomerism and dehydration. Diversity has been initiated by changing the Ar group on compound 28.^25^

**Scheme 8:**
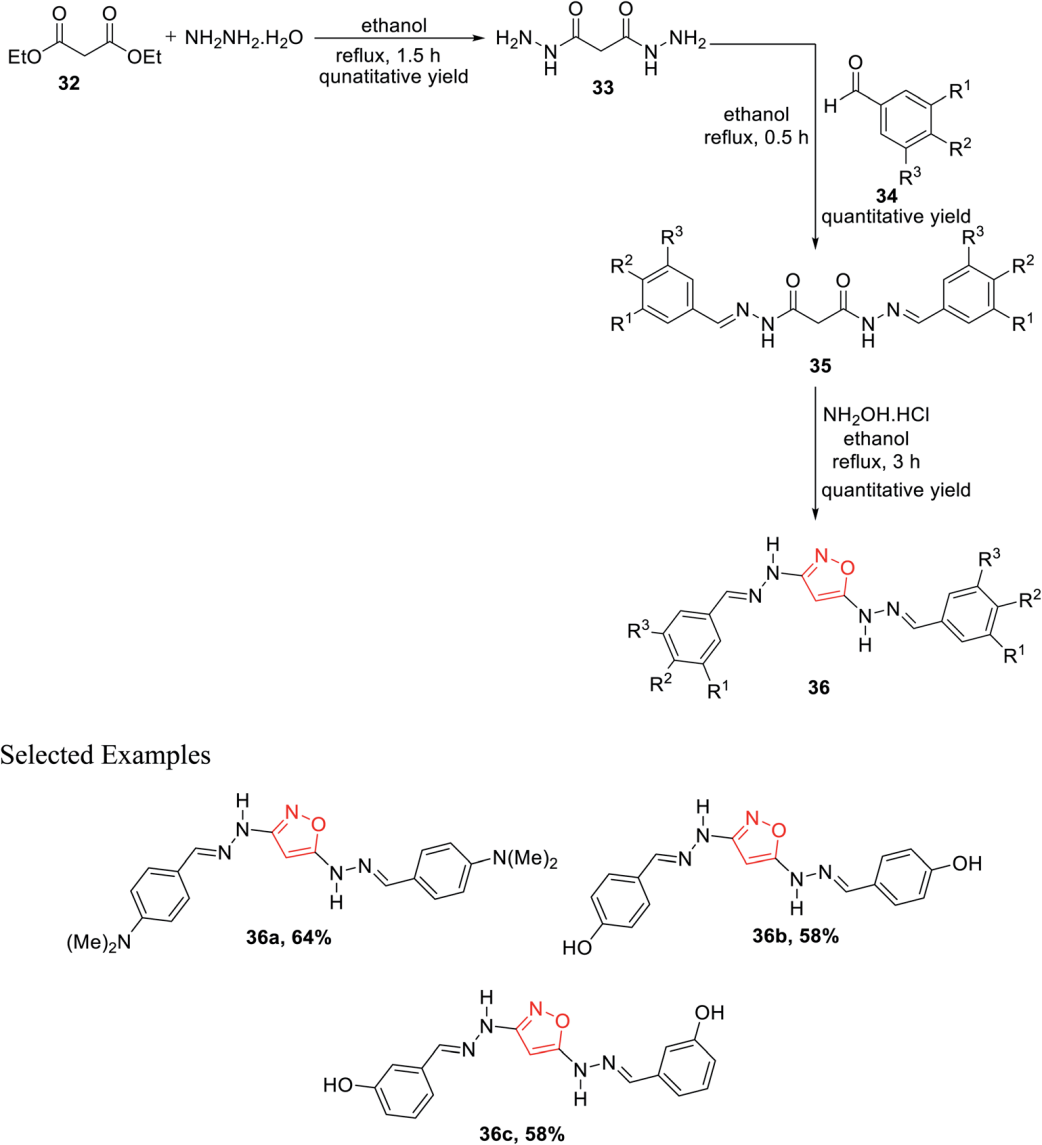
Preparation of 3,5-disubstituted isoxazole derivatives.

Subsequently, in 2011, Rai *et al.* showed a novel method for the synthesis of isoxazole derivatives and to evaluate their biological activity. To synthesize the privileged scaffold, malonodihydrazide 33 was taken as the starting material, which was synthesized by reacting diethylmalonate 32 with hydrazine hydrate in 1 : 2 molar ratio under reflux condition for 1.5 h using ethanol as the solvent. The product malonodihydrazide 33 was reacted with different aromatic aldehydes 34 in 1 : 2 molar ratio under refluxing condition for 30 min in ethanol solvent, which resulted in diaroylhydrazones 35. Further, diaroylhydrazones 35 were reacted with hydroxylamine hydrochloride under refluxing condition for 3 h in ethanol solvent, which led to the final product isoxazoles 36 (Scheme 8). Next, *in vitro* studies of compounds 36 were carried out for antibacterial and antioxidant activities. Compound 36 with *N*,*N*-dimethyl substituents at the *para* position showed dominant anti-lipid peroxidation, antioxidant, and higher antibacterial activity as compared to standard drugs.^26^

In the year 2013, Astudillo and his co-workers introduced a unique synthetic methodology for the generation of 3,5-disubstituted isoxazoles *via* the 1,3-dipolar cycloaddition reaction of an alkyne and a nitrile oxide. The nitrile oxide was obtained *in situ* by the conventional method of the reaction of an oxime and an oxidant.

**Scheme 9:**
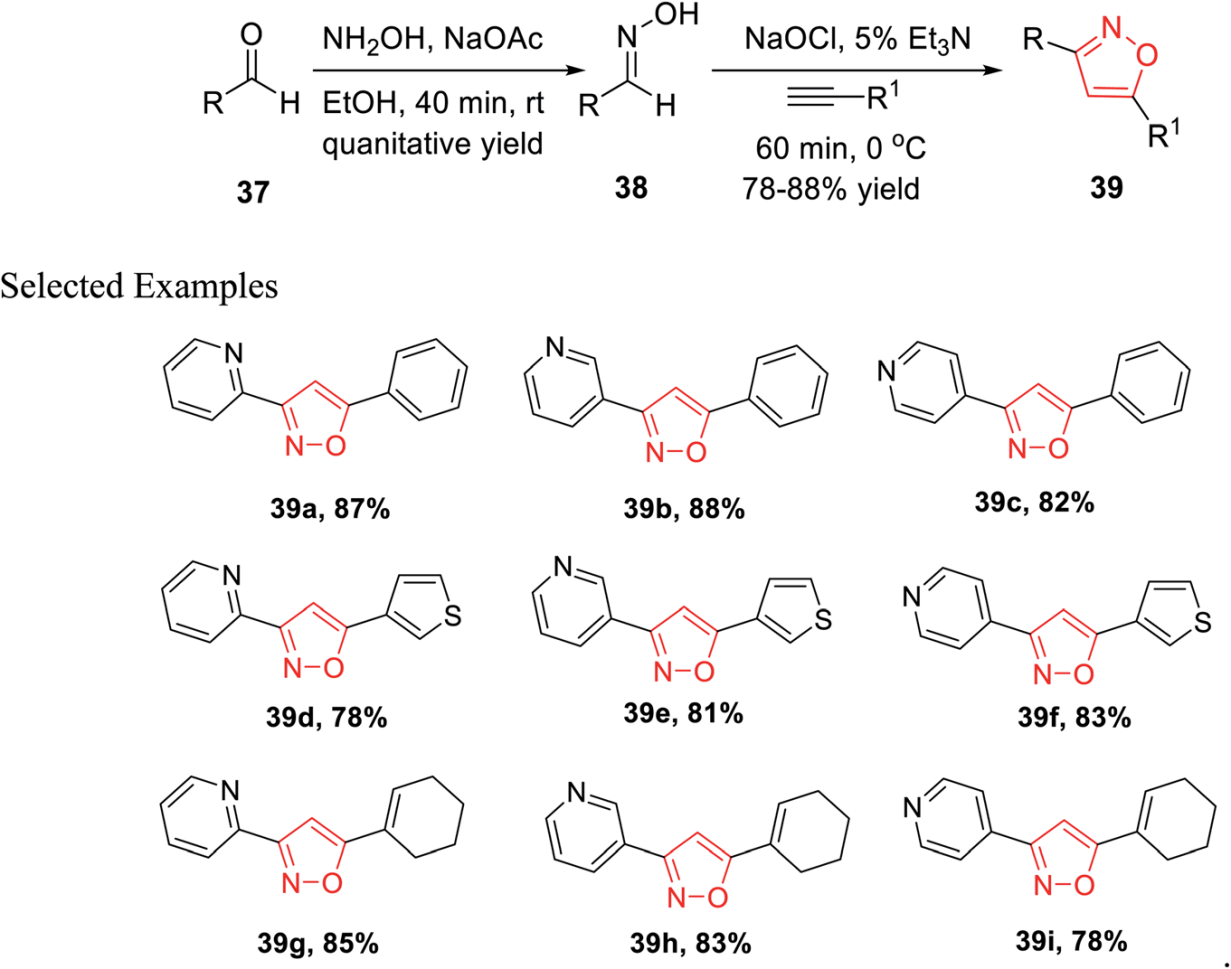
Synthesis of 3,5-disubstituted isoxazole.

**Fig. 4:**
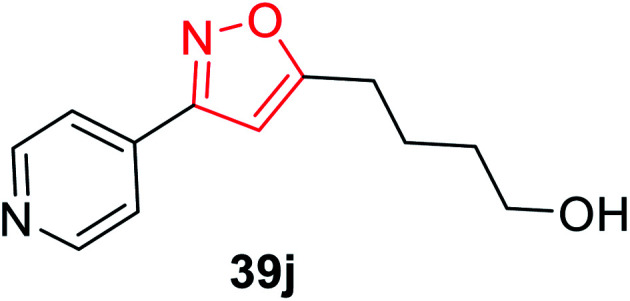
3,5-Disubstituted isoxazole derivative.

The synthesis of oxime 38 derivatives was achieved by the reaction of hydroxylamine and carbonyl compound 37 in the presence of sodium acetate and ethanol as the solvent for 40 min at room temperature (Scheme 9). The 3,5-disubstituted isoxazoles 39 were prepared by the reaction of oxime 38 and an alkyne in the presence of an oxidizing agent such as sodium hypochlorite and 5% triethylamine *via* the *in situ* generated nitrile oxide intermediate. The biological activity showed that the isoxazole derivative 39j (Fig. 4) acts as the acetylcholinesterase (AChE) inhibitor and the docking of the ligand-AchE complex indicates that derivative 39j was placed on the edge of the AChE active site. The main purpose of the AChE inhibitors is prohibiting the enzyme acetylcholinesterase from breaking down the neurotransmitter acetylcholine into choline and acetate, which resulted in an increase in both the level and duration of action of acetylcholine in the central nervous system. Compounds 39g, 39h, and 39i were scrutinized for the biological activity. They displayed the highest biological activity with the lowest IC_50_ values and they are lower than 200 μM, which are better than that of the other derivatives.^27^

**Scheme 10:**
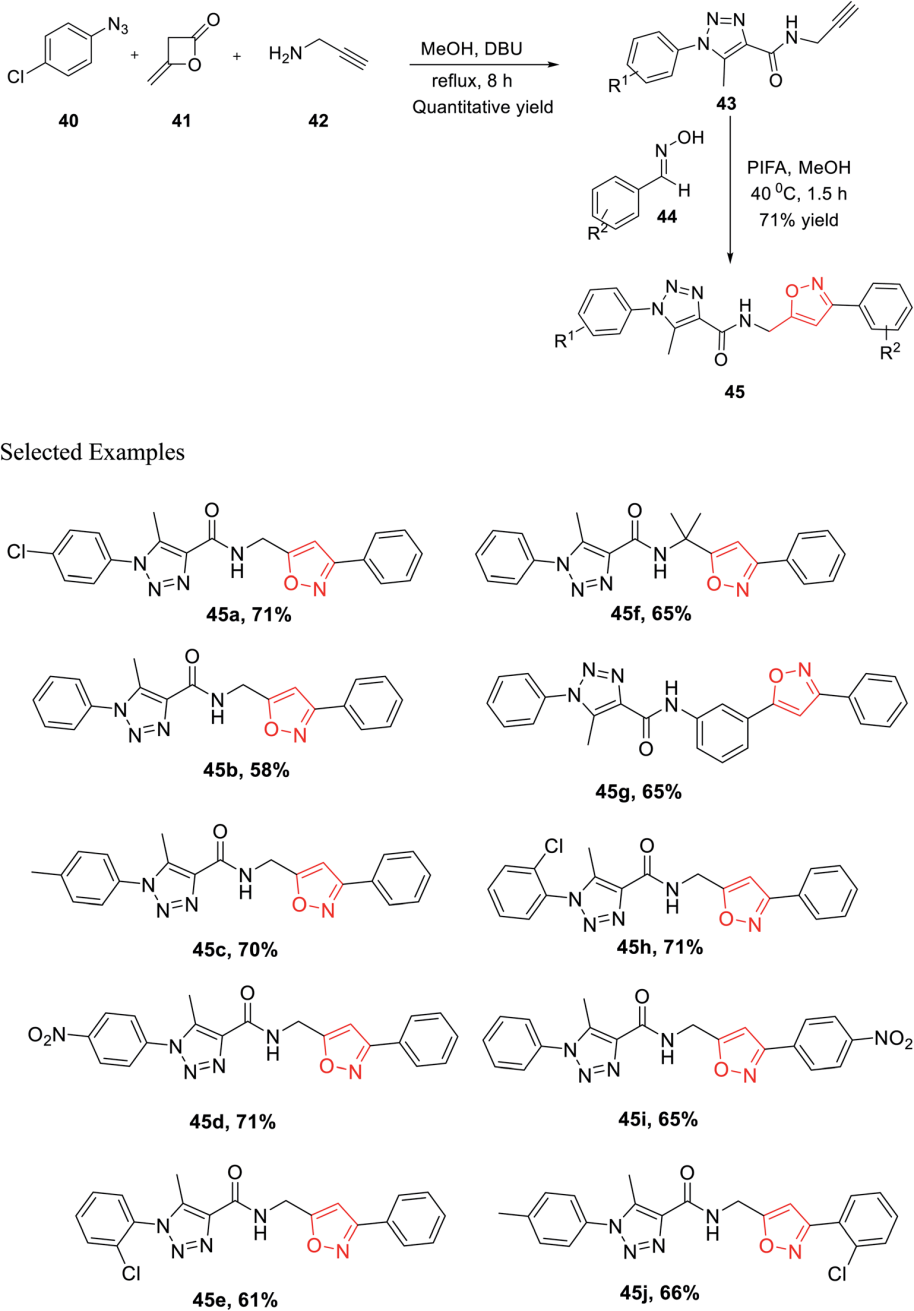
Chemoselective formation of bisfunctionalized hybrid 1,2,3-triazole-isoxazole molecules.

In the same year, Cai and his co-worker demonstrated a novel methodology for the generation of bisfunctionalized 1,2,3-triazole-isoxazole hybrid derivatives having peptide linkage. The methodology started with the synthesis of intermediate 43 by reacting azide 40, diketene 41, and propargyl amine 42 in a sealed tube under refluxing methanol solvent for 8 h. To get the final 1,2,3-triazole-isoxazole derivatives 45, the methanolic solution of intermediate 43 reacts with oxime 44 in the presence of phenyliodine bis(trifluoroacetate) for 1.5 h at 40 °C (Scheme 10). An impressive chemoselective pathway has been introduced to synthesize 1,2,3-triazole-isoxazole derivatives having peptide linkage using a completely metal-free catalyst (particularly not using Cu for triazole synthesis), maintaining the rule of “*Green Chemistry*” and atom economy up to the mark. Lipinski's rule-of-five study was executed on all the isoxazole derivatives 45(a–j) to assess the drug-likeness or to check the biological or pharmacological activity. It has been found that all the derivatives 45(a–j) follow all the parameters established by Lipinski. Subsequently, the outcomes propose that compounds 45(a–j) were very useful for to discover biological probes and drug leads.^28^

**Scheme 11:**
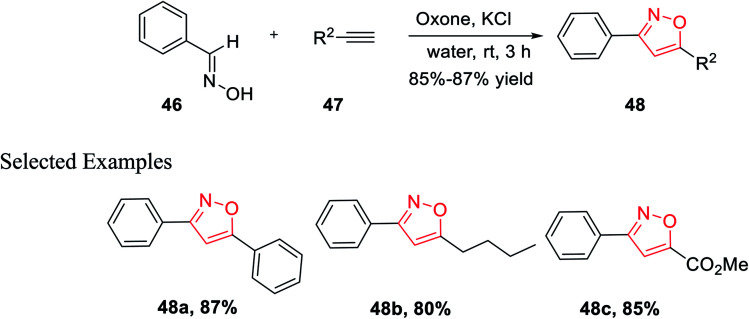
The cycloaddition of nitrile oxide from aldoxime to alkynes mediated by KCl and oxone.

In the year 2014, Yan and his co-workers developed an environmentally benign synthesis of isoxazoles using oxone (an oxidizing agent) in water medium. Oxone (2KHSO_5_-KHSO_4_-K_2_SO_4_) was used as an efficient oxidizing agent because it has excellent stability, high water solubility, easily transportable, non-toxic in nature, and less costly. The cycloaddition products 48 have been obtained by the reaction of aldoximes 46, alkenes 47 in presence of oxone in aqueous medium at room temperature for 3 h (Scheme 11). The mechanism of this reaction was involved as potassium chloride is oxidized into chlorine in water with the help of oxone, followed by the formation of nitrile oxide from the oxidation of aldoxime by the *in situ* developed hypochlorous acid. At last, 1,3-dipolar cycloaddition reaction occurred as nitrile oxide reacts with alkyne and resulted in the corresponding 3,5-disubstituted isoxazoles.^29^

**Scheme 12:**
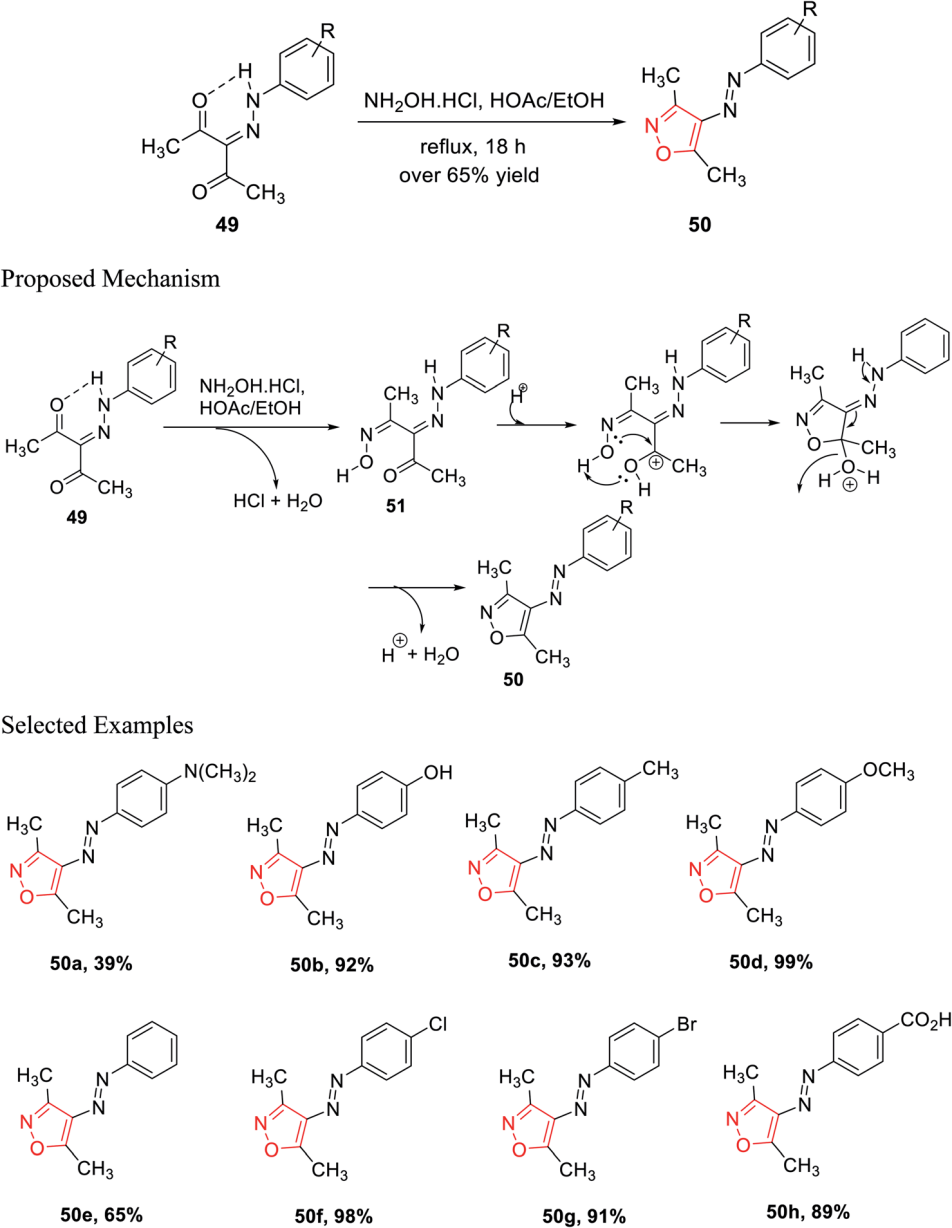
Synthesis of 3,4,5-trisubstituted isoxazole derivatives.

In the subsequent year, Scott and his group established a new method for the preparation of 3,4,5-trisubstituted isoxazole 50 derivatives from β-diketohydrazone 49. The carbonyl group of β-diketohydrazone 49 was stabilized by the existence of the hydrogen bond and its quasi-aromaticity^30*a*^ (Scheme 12).

Hence, the electrophilicity on the carbonyl group increased, which created outstanding reactivity difference between the two carbonyl groups; thus, regioselectivity was observed. By maintaining the reaction stoichiometry, water elimination from one carbonyl group was allowed; thus, hydroxylamine hydrochloride reacts with only one carbonyl group and gives the corresponding oxime as the intermediate. After that, the unreacted ketone group reacts with mono oxime intermediate 51, giving the corresponding isoxazole *via* the addition and displacement reactions. A series of isoxazole molecules have been synthesized using different R groups of 3-(2-(4-*R*-phenyl)hydrazinylidene)pentane-2,4-diones 49.

3-(2-(4-*R*-phenyl)hydrazinylidene)pentane-2,4-diones 49 was refluxed for 18 h with NH_2_OH·HCl, with a stoichiometric ratio of 1 : 1, in the presence of acetic acid, where ethanol was used as the solvent. Over 65% yields were obtained for each of the (*E*)-3,5-dimethyl-4-(*R*-phenyldiazenyl)isoxazole derivatives 50 and were recrystallized by the ethanol–water solution of varying compositions. The biological activity such as the cytotoxic effect of 3,4,5-trisubstituted isoxazoles 50 and the effect of 3,4,5-trisubstituted isoxazoles 50 on the interpretation of p21^WAF-1^, Bax, and Bcl-2 were observed. The biological study was performed using all the isoxazole derivatives and integrating them into leukemia HL-60 cells culture. Interestingly, compounds 50c and 50f displayed lower IC_50_ values that are 95.4 μM and 85.6 μM, respectively. Both the molecules exhibit the maximum cytotoxic action toward HL-60 cells.^30*b*^

**Scheme 13:**
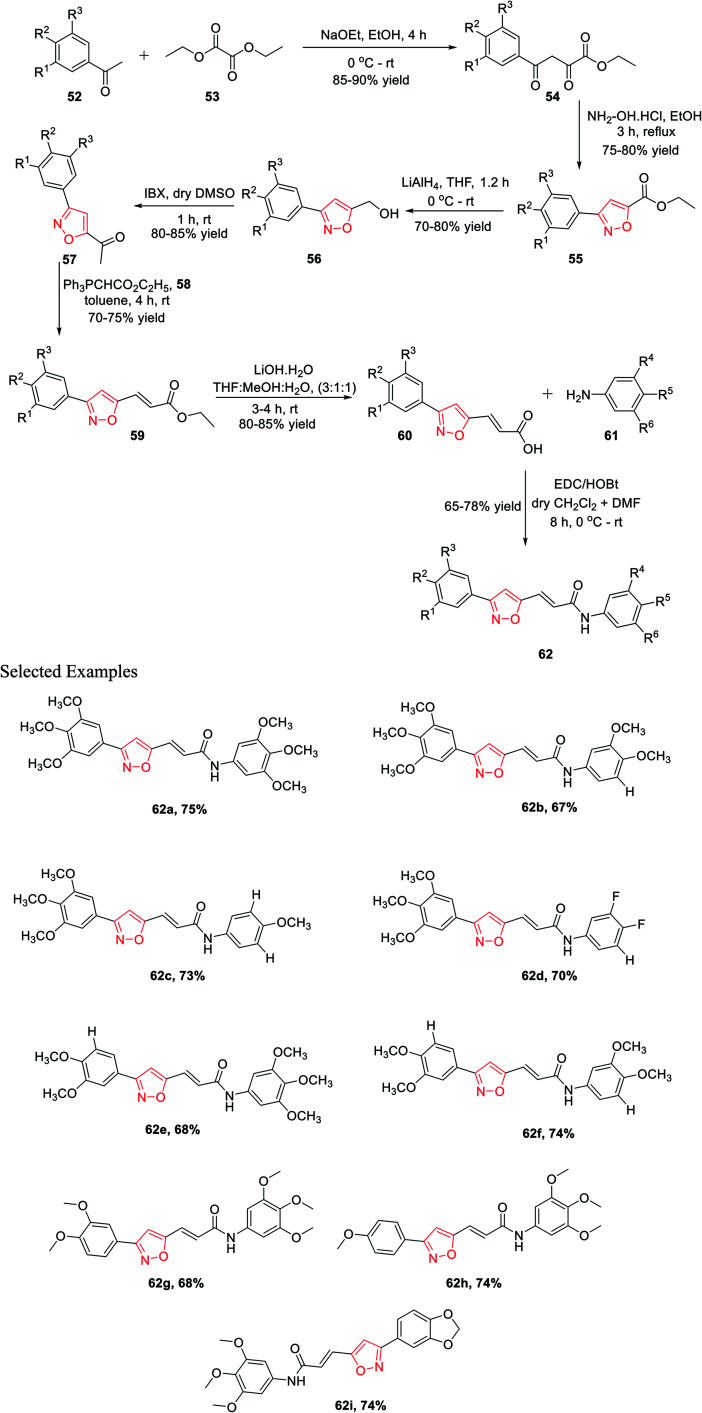
Synthesis of isoxazole-linked arylcinnamides moieties.

In 2015, Kamal and his co-workers have accomplished a new synthetic method for isoxazole connected with the arylcinnamide moiety.^31^ Isoxazole-linked arylcinnamide derivatives are an important scaffold; hence, an effort was made to unravel their cytotoxic potential (Scheme 13). The synthesis was started with the condensation of diethyl oxalate 52 and different acetophenones 53 in the presence of sodium ethoxide to obtain compound 54. The reaction of intermediate 54 with hydroxylamine hydrochloride in refluxing ethanol solution resulted in isoxazole-linked esters 55 in good yield. The further reduction of the ester moiety in compound 55 using lithium aluminium hydride yielded 56. Subsequently, the oxidation of isoxazole-linked alcohols 56 by 2-iodoxybenzoic acid (IBX) in dry DMSO introduced the ketone moiety in isoxazoles 57. The ketone-linked isoxazoles 57 were reacted with Ph_3_PCHCO_2_C_2_H_5_ (C2-Wittig reagent) 58 in the presence of toluene solvent to afford α,β-unsaturated esters 59. Next α,β-unsaturated esters 59 underwent base-mediated hydrolysis and yielded the parallel carboxylic acids 60. The synthesized isoxazole containing carboxylic acids 60 were coupled with different anilines 61 using EDCI/HOBt as the coupling agents to obtain the corresponding isoxazole connected with arylcinnamide moiety 62. These molecules 62(a–i) were assessed for their capability to prevent the progress of several human cancer cell lines, for example A549, HeLa, MDA-MB231, and DU-145, among which some displayed considerable cytotoxic effects. Compounds 62a, 62g, 62h, and 62i displayed moderate cytotoxic activity. These compounds showed the lowest IC_50_ values compared to the other derivatives, with IC_50_ values in the range of 2.9–5.4 μM for the HeLa cells. Similarly, they exhibit the lowest IC_50_ values for DU-145, MDA-MB231, and A549.^31^

**Scheme 14:**
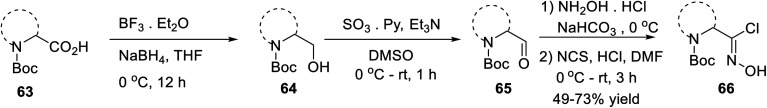
Synthesis of *N*-Boc-masked chloroxime.

In 2016, Mykhailiuk and his group introduced the base-mediated metal-free synthesis of aminoisoxazoles at the multigram scale. Aminoisoxazoles are very useful for peptidomimetics and drug discovery. The (3 + 2)-cycloaddition reaction was the main reaction where the *in situ* produced nitrile oxide reacted with alkyne/enamines regioselectively (Scheme 14).

The first step of the synthetic methodology was started with the reduction of the carboxylic group of *N*-Boc-masked amino acid 63 using NaBH_4_ from 0 °C to rt for 12 h and yielded the corresponding *N*-Boc amino alcohol 64. The obtained *N*-Boc amino alcohol 64 was oxidized by SO_3_·Et_2_O in the presence of DMSO solvent from 0 °C to rt, which led to the corresponding aldehyde 65. The resulting aldehyde furnished the oxime by the initial treatment of hydroxylamine hydrochloride in the presence of moderately basic NaHCO_3_. The so-formed oxime underwent chlorination with *N*-chlorosuccinimide in DMF from 0 °C to rt for 3 h, which yielded *N*-Boc-masked chloroxime 66.

**Scheme 15:**
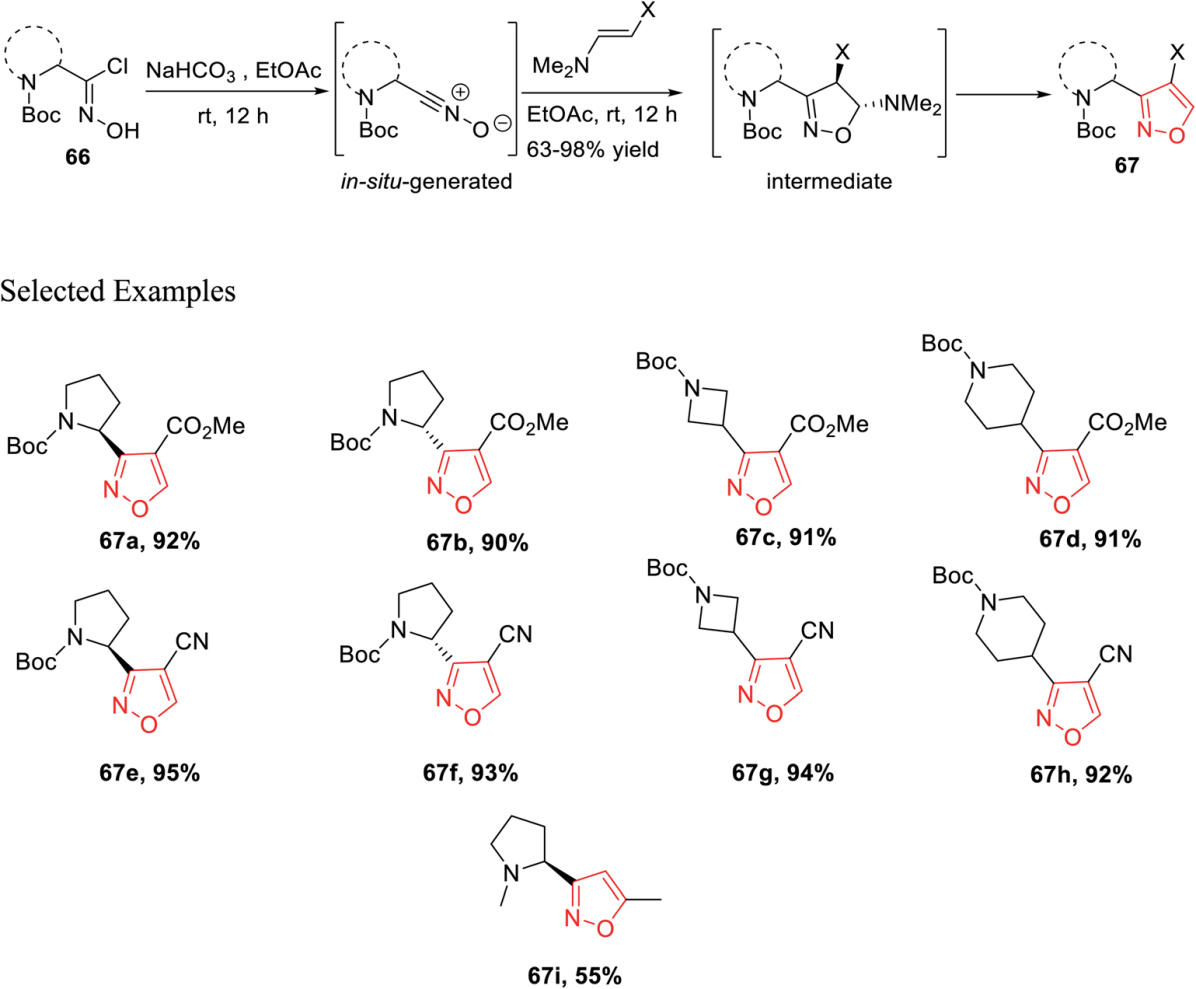
Synthesis of *N*-Boc-masked isoxazole derivatives.

Isoxazoles 67 were synthesized from the reaction of alkynes/enamines and *in situ* created nitrile oxides (Scheme 15). The reaction of *N*-Boc-masked chloroxime 66 with a mild base such as NaHCO_3_ or Et_3_N at room temperature or 0 °C was obtained corresponding to the *in situ* nitrile oxide in ethyl acetate medium. Then, *in situ* generated nitrile oxide was reacted with alkyne/enamine in the same ethyl acetate medium at the room temperature for 12 h, which yielded an intermediate that leads to the final product isoxazole 67 at the multigram scale. Compound 67i is commercially available as the ABT-418 drug, which is recognized as a nootropic agent along with both neuroprotective and anxiolytic effects. Compound 67i was also investigated for the treatment of both Alzheimer's disease and attention deficit hyperactivity disorder (ADHD) which is a mental and behavioral disorder characterized by inattention. Previously, the reported synthesized protocol furnished only 7.3% yield of ABT-418. However, herein the potential drug candidate 67i was synthesized with excellent overall yield of 55%.^32^

**Scheme 16:**
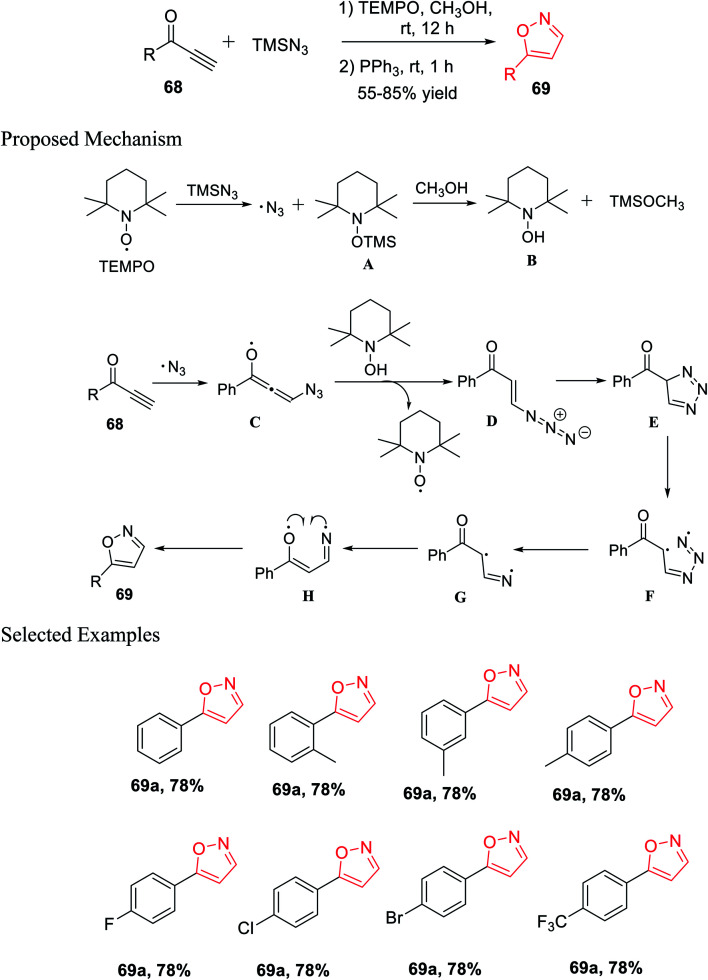
Synthesis of 5-substituted isoxazole using the TEMPO catalyst.

Again, in the same year, Pan and his co-workers introduced the unique and effective synthesis of 5-substituted isoxazole from TMSN_3_ and propargylic ketones using TEMPO as the catalyst. This synthetic procedure provided different valuable 5-substituted isoxazoles from easily accessible TMSN_3_ and propargylic ketones with outstanding yields. Initially, propargylic ketones 68 were reacted with TMSN_3_ in the presence of TEMPO and methanol as the solvent at room temperature for 12 h. Subsequently, PPh_3_ was added and the reaction mixture was stirred for another 1 h at room temperature, which resulted in 5-substituted isoxazoles 69 (Scheme 16).^33^

A possible mechanism was proposed for the preparation of 5-substituted isoxazoles. Initially, TEMPO reacted with TMSN_3_, which gave the azido free radical along with the creation of intermediate A. The TEMPO-coupled TMS compound A was reacted with methanol and produced TEMPOH B and CH_3_O-TMS. The azido free radical afterward attacked the alkyne and produced radical intermediate C. After radical C reacted totally with the TEMPOH compound B, intermediate D was obtained, which was confirmed by the HRMS study, with the revival of the catalyst. Hence, the intramolecular cycloaddition reaction of azide-alkene took place and yielded triazole E. The obtained triazole E experienced homolytic cleavage to produce the radical intermediate F, which freed N_2_ and generated the intermediate radical G. Intermediate radical G undergoes one-electron transfer and gave radical compound H, which finally underwent intramolecular radical coupling to furnish 5-substituted isoxazole 69.

**Scheme 17:**
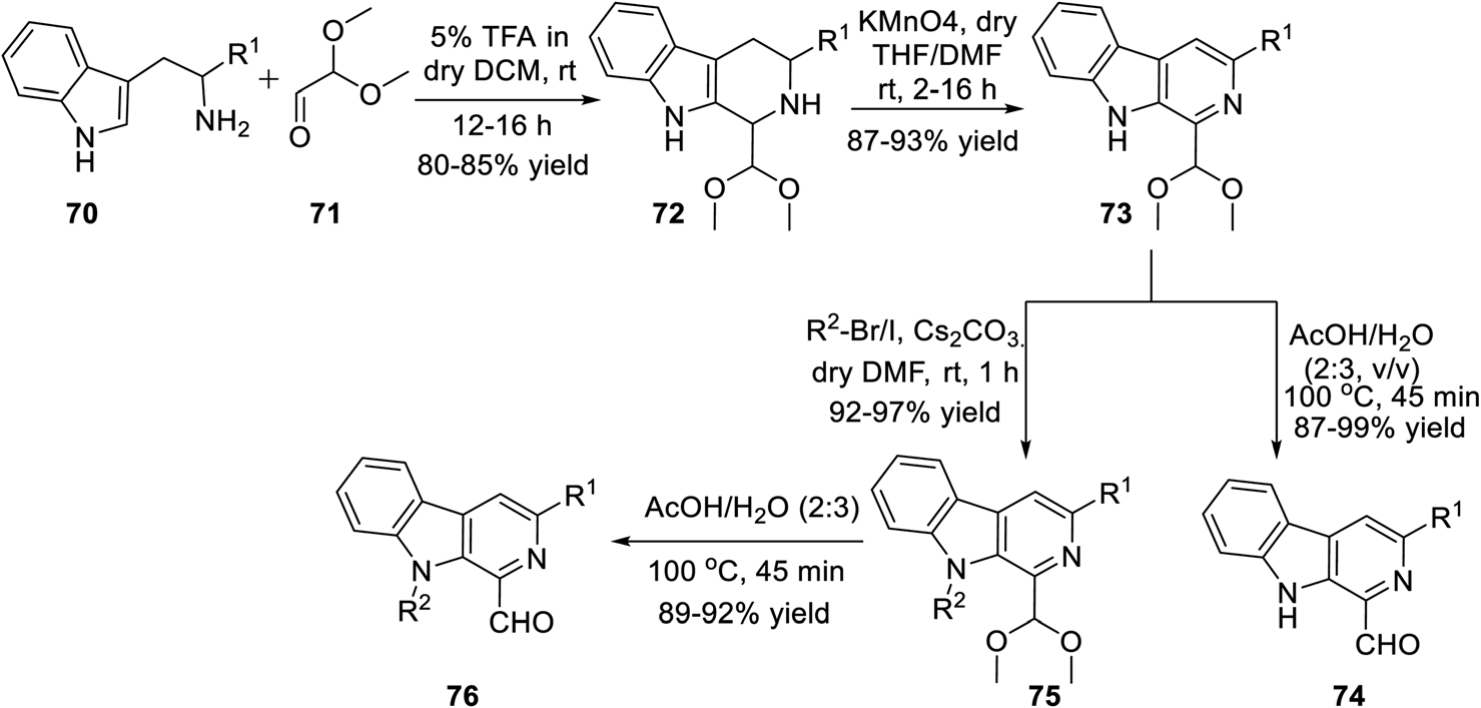
Synthetic procedure of 1-formyl-9*H*-pyrido[3,4-*b*]indoles and its *N*-substituted analogue.

Subsequently, Singh's group reported a unique method for the regioselective synthesis of a scaffold containing both β-carboline and isoxazole together *via* the 1,3-dipolar cycloaddition reaction. To synthesize the desired product, l-tryptophan ester 70 was chosen as the primary precursor. The l-tryptophan ester 70 underwent the sequence of reactions to obtain 1-formyl-9*H*-β-carbolines 74 and its *N*-alkylated analogue 76 (Scheme 17).

**Scheme 18:**
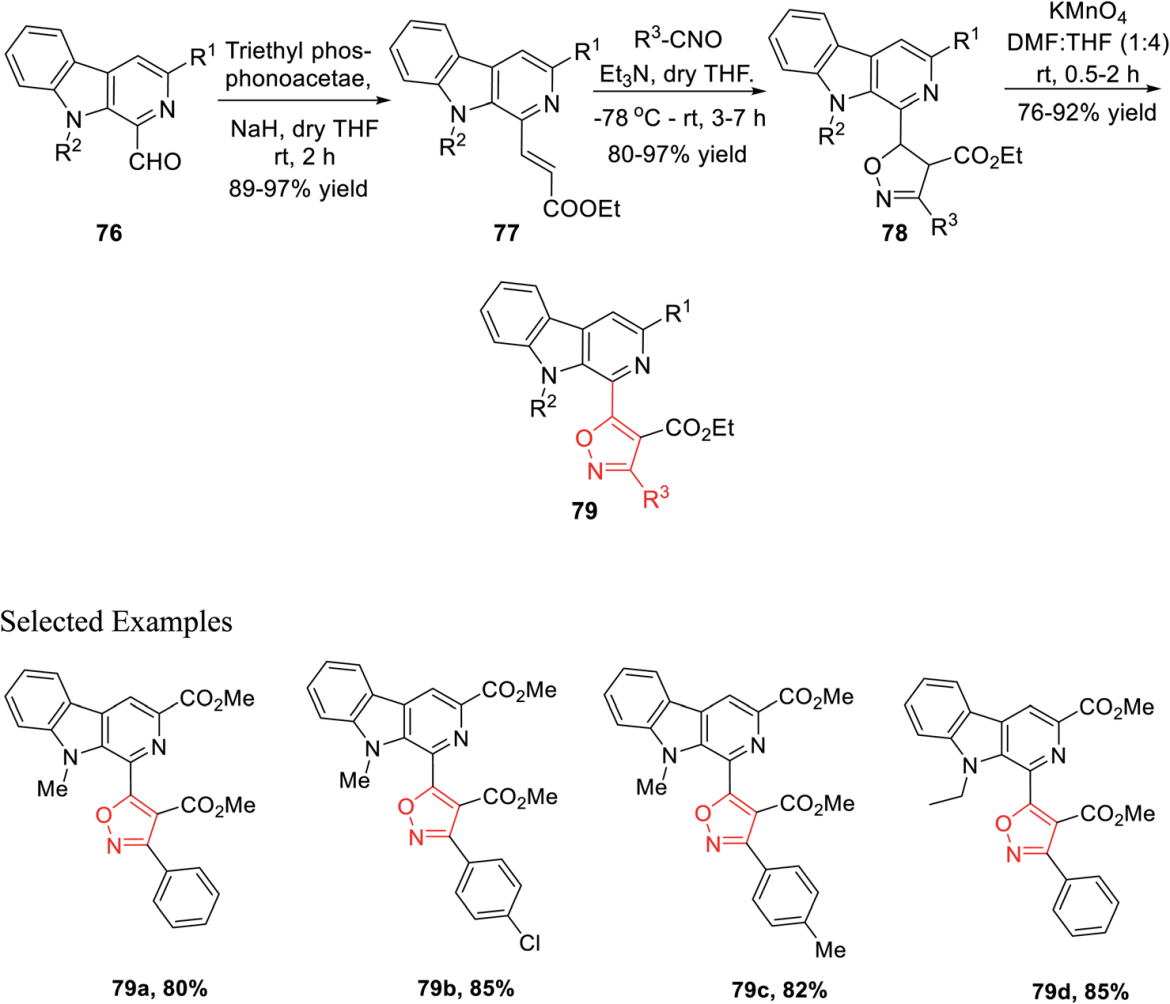
Metal-free multi-step synthesis of β-carboline-based 3,4,5-trisubstituted isoxazoles.

The next sequence of reaction involved the formation of the isoxazole moiety onto the β-carboline scaffold. Initially, *N*-substituted-β-carbolines 76 were reacted with triethyl phosphonoacetate in dry THF and resulted in the Wittig^34*a*^ product 77. After that, the nitrile oxides produced *in situ* were reacted with the Wittig product 77 in the presence of Et_3_N as the base in anhydrous THF solvent, finally producing the corresponding isoxazoline products 78. Finally, isoxazoline derivatives 78 were oxidized by KMnO_4_ in DMF and dry THF mixture (1 : 4) at room temperature, which furnished β-carboline-based isoxazoles 79 (Scheme 18).

**Scheme 19:**
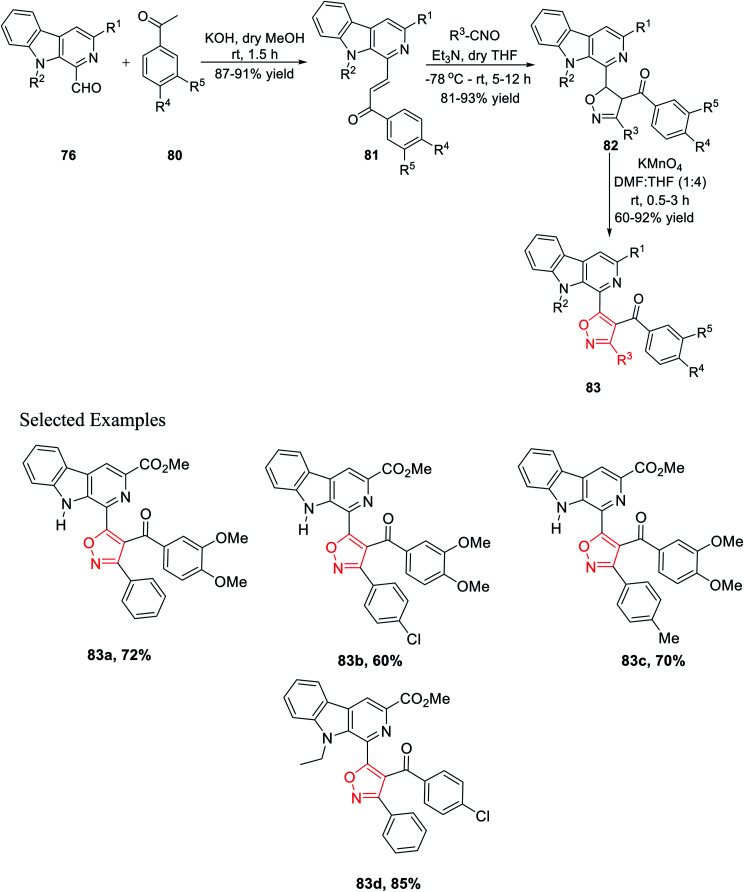
Formation of β-carboline-linked isoxazole derivatives with chalcone as the prototype.

Again, another β-carboline-based chalcone derivatives 77 was synthesized by the Claisen–Schmidt condensation from 1-formyl-pyrido[3,4-*b*]indole molecules 76 with various acetophenone derivatives containing both electron donating clusters and electron withdrawing clusters. Fascinatingly, β-carboline-linked chalcones 77 (dipolarophile) were reacted effortlessly with different nitrile oxides in the presence of dry THF and Et_3_N at −78 °C, which yielded the desired isoxazoline derivatives 78. Hence, the isooxazoline derivatives 78 were underwent the same oxidation reaction in the presence of KMnO_4_ in dry THF and DMF mixture (4 : 1) at room temperature and furnished the β-carboline-linked isoxazoles 79 with decent to outstanding productivity (Scheme 19).

**Scheme 20:**
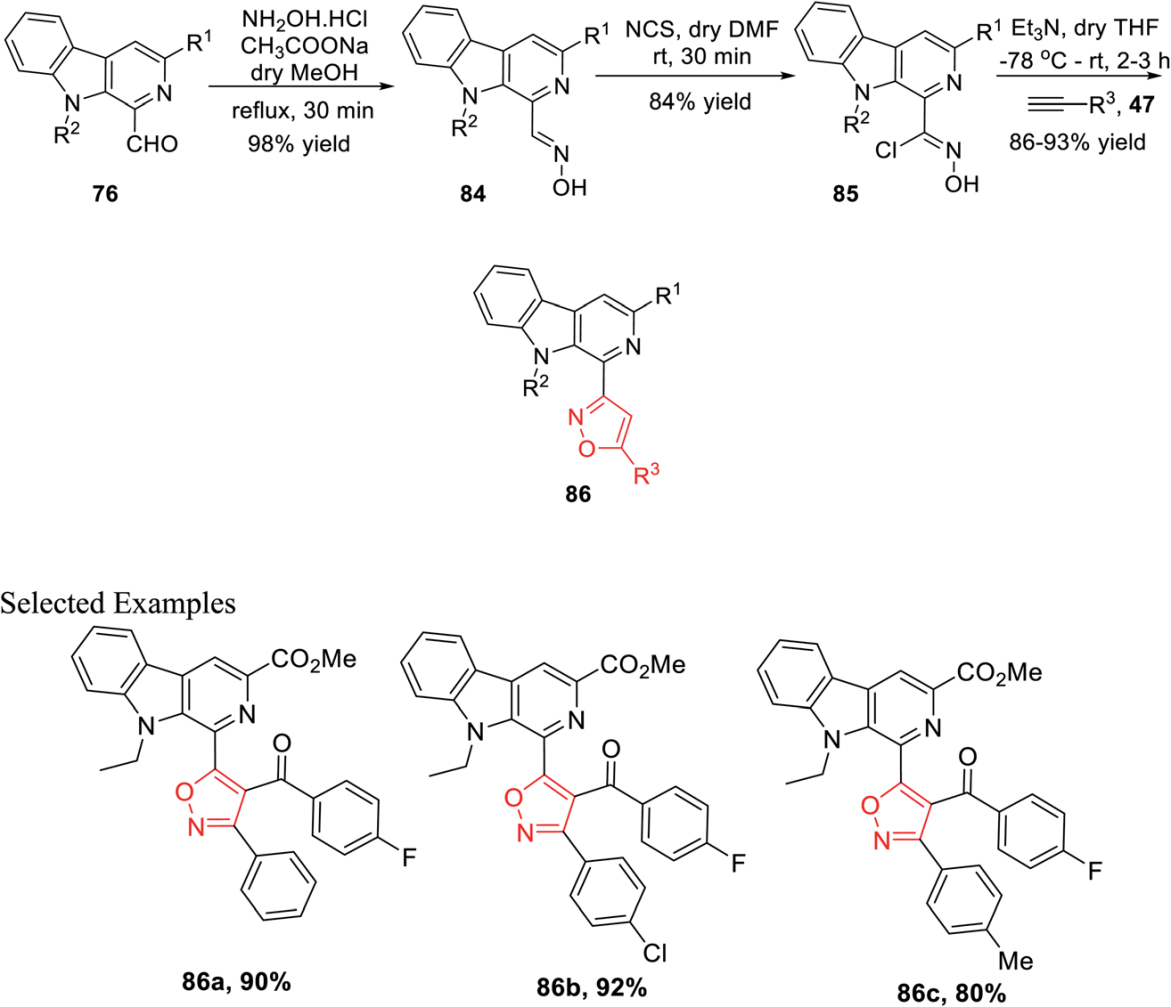
Formation of the β-carboline-(C-3) isoxazole derivative *via* the oxime intermediate.

After successfully synthesizing β-carboline-linked isoxazole derivatives, another different kind of β-carboline-linked isoxazole derivatives was synthesized *via* the formation of the oxime intermediate. The *N*-alkylated analogue 76 was reacted with NH_2_OH·HCl in dry methanol to furnish the corresponding oximes 84. Further, oximes 84 were reacted with *N*-chlorosuccinimide (NCS) in dry DMF at ambient temperature, which yielded the corresponding hydroxyimoyl chlorides 85. Finally, hydroxyimoyl chlorides 85 were reacted with various alkynes 47 (dipolarophiles) *via* the *in situ* formation of nitrile oxide using Et_3_N as the base in anhydrous THF at −78 °C, which afforded the desired products β-carboline-linked isoxazoles 86 (Scheme 20).^34*b*^

**Scheme 21:**
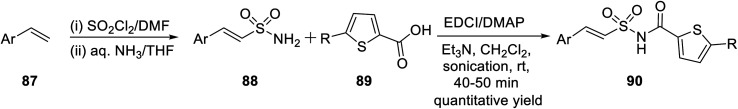
Synthetic procedure for the synthesis of arylethenesulfonyl thiophene-2-carboxamides.

In the year 2017, Padmavathi *et al.* reported a unique method to synthesize isoxazole derivatives having sulphonamide linkage with thiophene. The reaction took place *via* the routine 1,3-dipolar cycloaddition reaction employing the green approach. To synthesize isoxazole scaffolds having amide linkages with thiophene moiety 93, synthetic intermediate thiophenylamidosulfonyl styrenes 90 were required to be synthesized. Initially, styrene 87 was reacted with sulfuryl chloride in DMF solvent, followed by treatment with aqueous ammonia in THF medium to get arylethenesulfonamide 88. Subsequently, compound 88 was reacted with thiophene-2-carboxylic acid 89 using 1-ethyl-3-(3-dimethylaminopropyl)-carbodiimide (EDCI) as the coupling agent along with *N*,*N*-dimethylaminopyridine (DMAP) and Et_3_N as the base, which yielded (*E*)-*N*-(arylethenesulfonyl)thiophene-2-carboxamide 90 (Scheme 21).

**Scheme 22:**
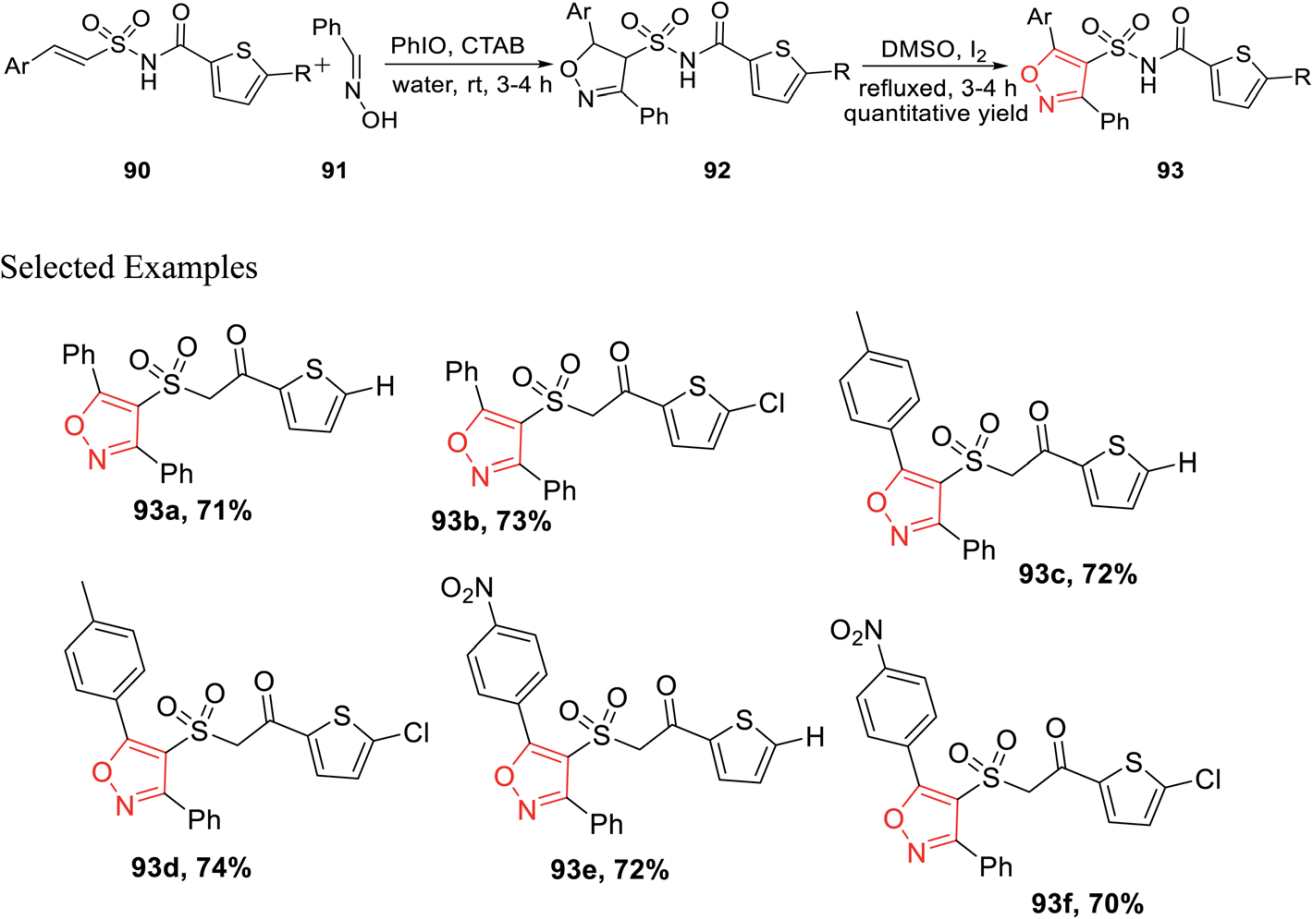
Synthetic pathway for isoxazole derivatives having the sulphonamide linkage with thiophene.

Subsequently, the synthesized (*E*)-*N*-(arylethenesulfonyl)thiophene-2-carboxamide 90 was reacted with araldoxime 91 in the presence of CTAB and iodosobenzene in water medium at ambient temperature, which furnished *N*-((5-aryl-3-phenyl-4,5-dihydroisoxazol-4-yl)sulfonyl)thiophene-2-carboxamide 92. Finally, molecule 92 was oxidized as well as aromatized to *N*-((5-aryl-3-phenylisoxazol-4-yl)sulfonyl)thiophene-2-carboxamide 93 with the help of I_2_ in DMSO (Scheme 22).^35^

Compounds 93e and 93f demonstrated outstanding antibacterial action on *B. subtilis*, which was higher than the typical drug chloramphenicol. Subsequently, the same two derivatives exhibit excellent antifungal action, which is better than that of the typical drug, ketoconazole. Again, *in vitro* study suggests that 93e and 93f act as efficient antibacterial agents toward *B. subtilis* and antifungal agents toward *A. niger*. The existence of electron withdrawing groups on the aromatic ring increased the activity.

**Scheme 23:**
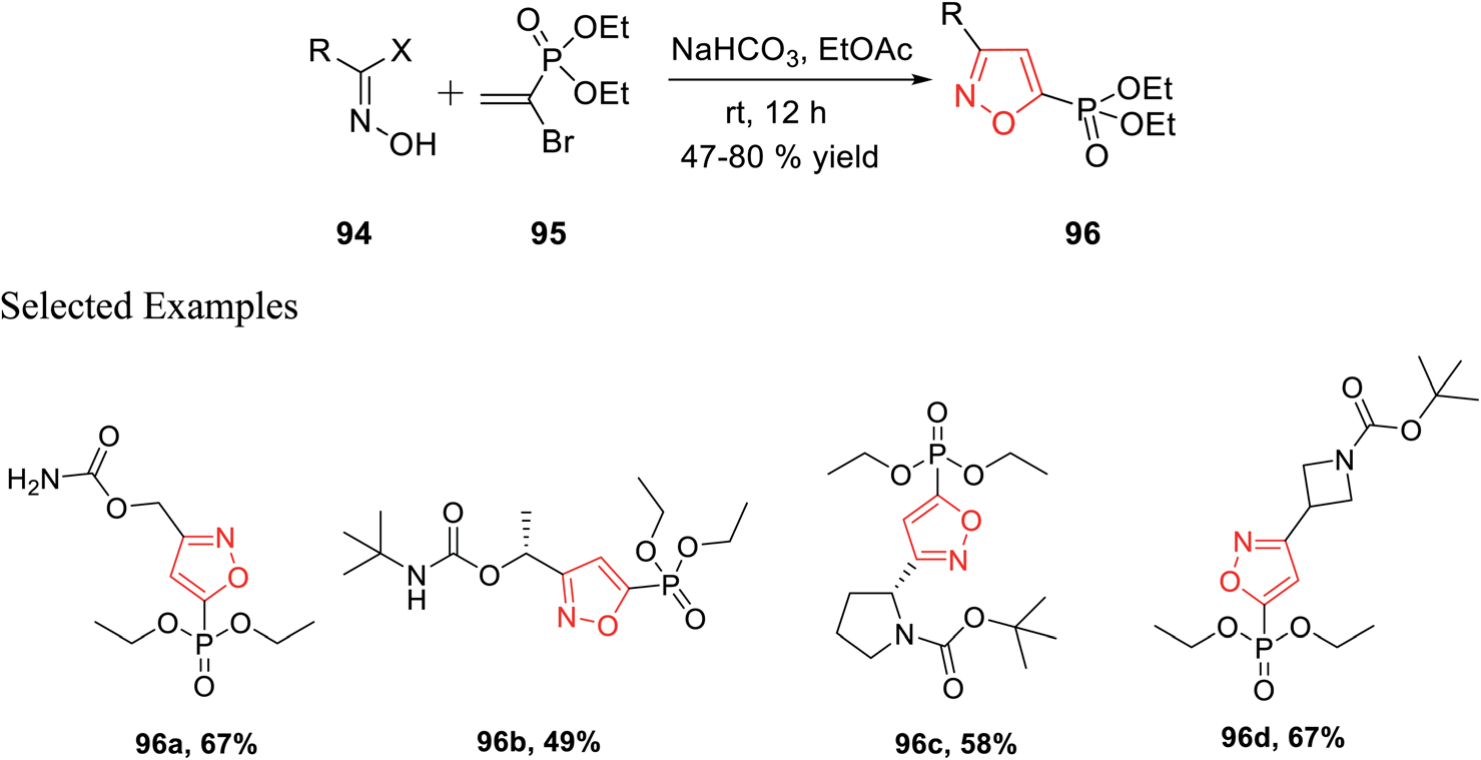
Regioselective synthesis of 3,5-disubstituted isoxazoles.

In 2018, Grygorenko *et al.* accomplished a new method to synthesize phosphonate-linked isoxazole derivatives through the (3 + 2) cycloaddition reaction. The (3 + 2) cycloaddition reaction took place regioselectively *via* various types of nitrile oxides and different types of dipolarophile. Initially, the regioselective synthesis of 3,5-disubstituted isoxazoles was attempted under mild basic condition (NaHCO_3_) at ambient temperature *via* the reaction of hydroxyimoyl halides and dipolarophile. Unfortunately, a mixture of 3,5-disubstituted isoxazoles and 3,4-disubstituted isoxazoles was obtained. The deciding factor of the regioselectivity of the substituted isoxazole is the leaving group of dipolarophile in the (3 + 2) cycloaddition reaction. Thus, diethyl-1-bromovinyl phosphonate 95 as the dipolarophile was designed to obtain only 3,5-disubstituted isoxazoles 96 regiospecifically *via* the reaction of oxime 94 with compound 95 under moderately basic condition (NaHCO_3_) at room temperature (Scheme 23).

**Scheme 24:**
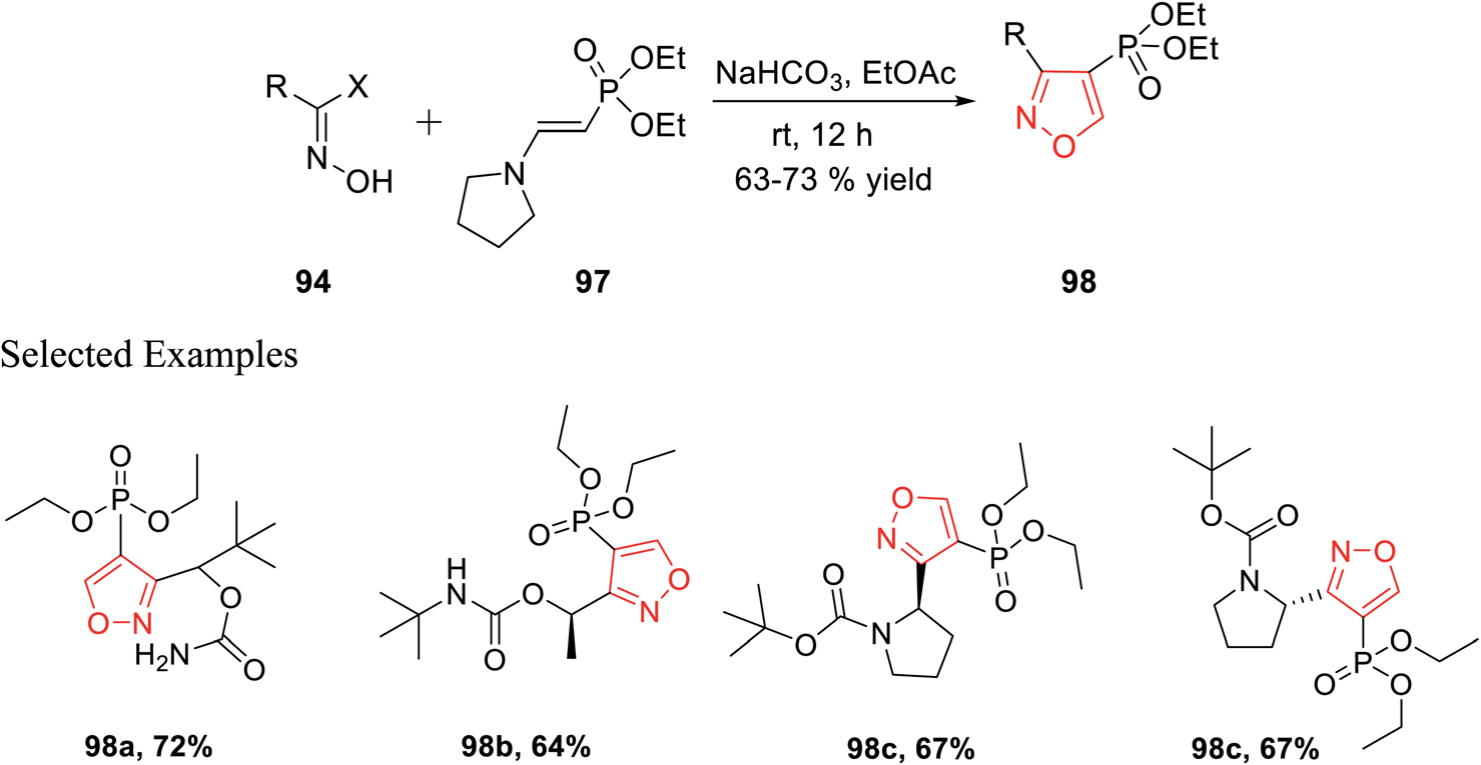
Regioselective preparation of 3,4-disubstituted isoxazoles.

Similarly, diethyl[2-pyrrolidin-1-ylethenyl]phosphonate 97 was prepared as the dipolarophile to obtain 3,4-disubstituted isoxazoles 98*via* the reaction of oxime 94 with compound 97 in the presence of moderately basic condition (NaHCO_3_) at room temperature (Scheme 24).

Both the reactions took place *via* the *in situ* formation of nitrile oxide. The synthesized phosphonate-linked isoxazole derivatives functioned as a suitable synthetic NDMD receptor.^36^

**Scheme 25:**
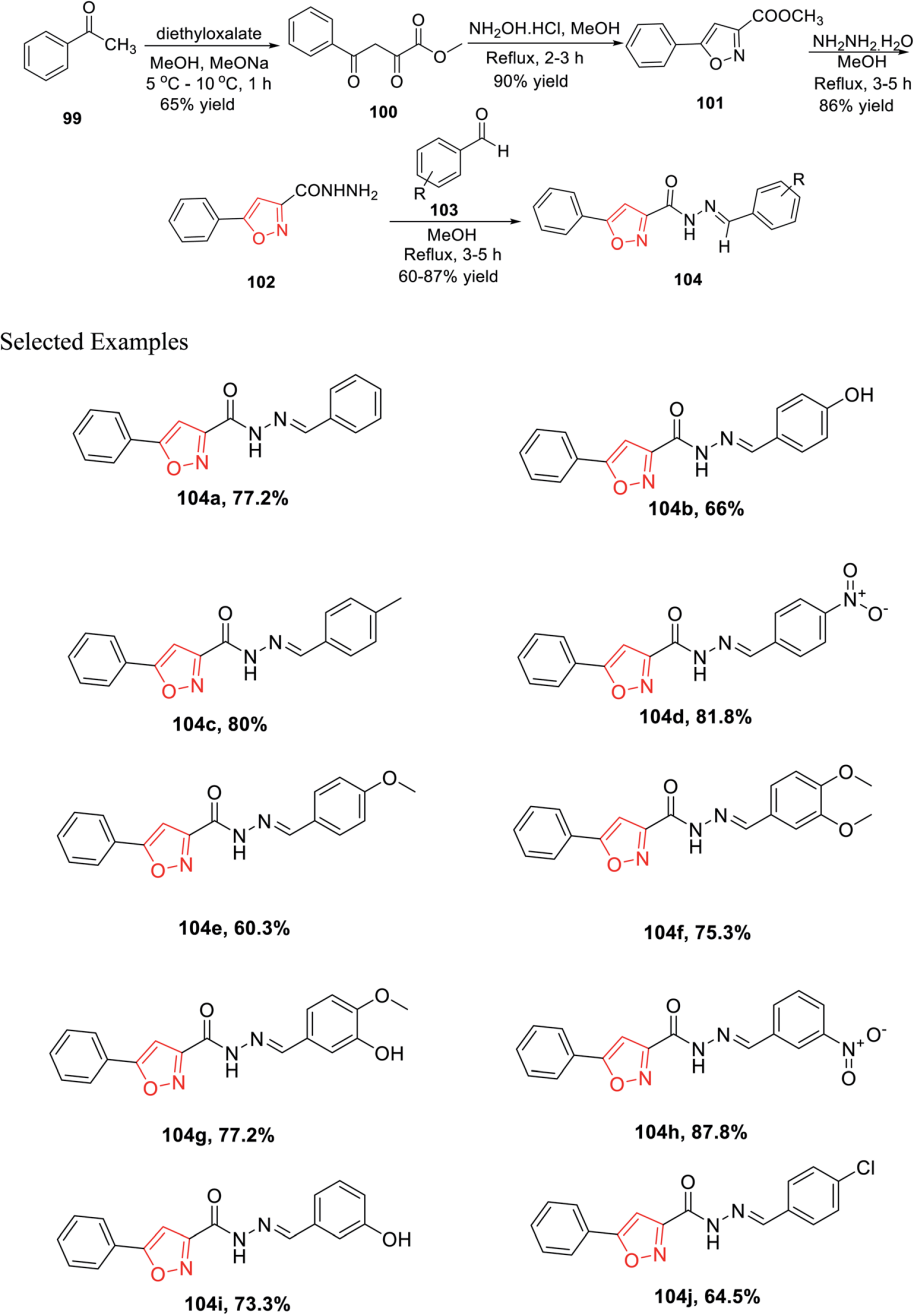
Synthesis of 3,5-disubstituted isoxazole scaffolds.

In 2019, Mishra and his group introduced a synthetic method for novel 3,5-disubstituted isoxazole scaffolds, which can be used as a possible anti-Parkinson agent. Initially, acetophenone 99 was reacted with diethyl oxalate in the presence of methanol and sodium methoxide at low temperature, which furnished methyl 2,4-dioxo-4-phenylbutanoate 100. Subsequently, intermediate 100 was cyclized with the help of NH_2_OH·HCl in refluxing methanolic condition for 2–3 h and yielded methyl 5-phenylisoxazole-3-carboxylate 101. Afterward, isoxazole 101 was reacted with hydrazine hydrate in refluxing methanolic condition for 3–5 h, which yielded 5-phenylisoxazole-3-carbohydrazide 102. Finally, the dehydration reaction took place with the reaction of 102 and functionalized benzaldehydes 103 in refluxing methanol solvent for 3–4 h and afforded functionalized *N*′-benzylidene-5-phenylisoxazole-3-carbohydrazide derivatives 104 (Scheme 25).

Intriguingly, monoamine oxidase (MAO) enzymes play a critical role in neurological disorders. Hence, functionalized *N*′-benzylidene-5-phenylisoxazole-3-carbohydrazide derivatives 104(a–j) were planned, synthesized, and utilized equally for MAO-A and MAO-B inhibition with the help of Amplex Red assays. The effect of the electron withdrawing groups and donating groups at the 4-position and/or the 3-position of the phenyl ring result in the difference in the case of MAO-B inhibition. Among all the derivatives, 104c displayed excellent MAO-B inhibition activity with an IC_50_ value of 0.0053 ± 0.0003 μM. Compounds 104g and 104b exhibit lower IC_50_ values than other derivatives. Compounds such as 104e and 104f having the methoxy group and 104j having the chloro group at the 4-position as the electron donating group showed less activity.^37^

**Scheme 26:**
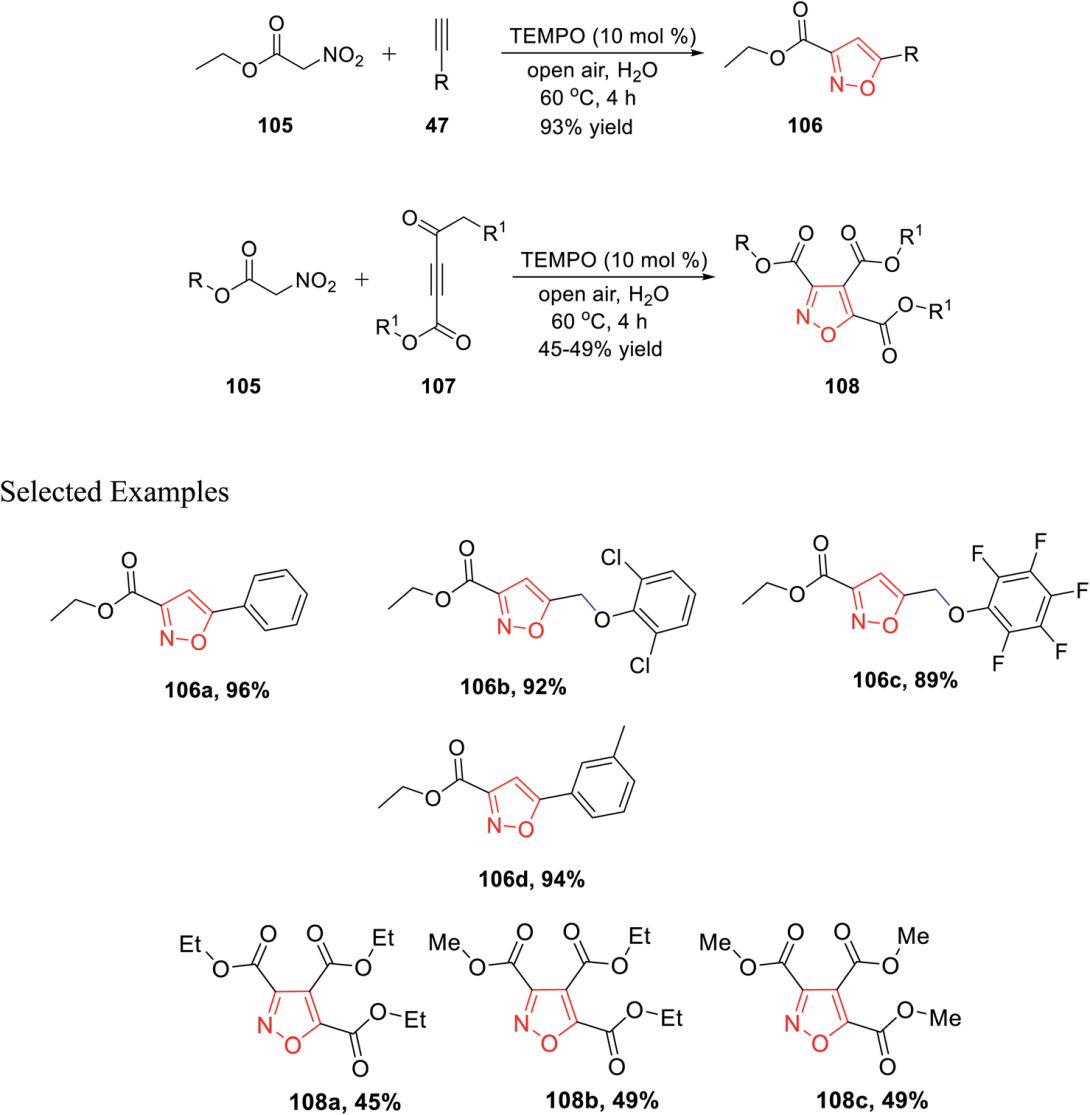
Preparation of highly substituted isoxazoles *via* TEMPO catalysis in air oxidation.

In 2019, Praveen *et al.* demonstrated a new unique method of TEMPO-catalyzed synthesis of substituted isoxazole derivatives *via* air oxidation. The use of water as the solvent along with TEMPO as the green oxidant fulfills the criteria of green synthetic procedures. Initially, gram-scale synthesis has performed by reacting ethyl nitroacetate 105 with phenylacetylene 47 in the presence of TEMPO, water, and in open air to give 3,5-disubstituted isoxazole 106 with an excellent yield of 93%. Afterward, the library synthesis was performed by introducing different functionalized nitroacetate 105 and different types of alkyne 107. Functionalized nitroacetate molecules 105 were reacted with alkyne molecules 107, which resulted in 3,5-disubstituted isoxazoles 108 under the same condition using water as the solvent in open air with the TEMPO catalyst (Scheme 26).^38^

Further, in the same year, Baranov and his group reported a new method to synthesize 3,4,5-trisubstituted isoxazole derivatives from aromatic aldehyde and nitroacetic esters *via* the intermediate alkyl-5-hydroxy-6-oxo-4-aryl-6*H*-1,2-oxazine-3-carboxylate derivatives.

**Scheme 27:**
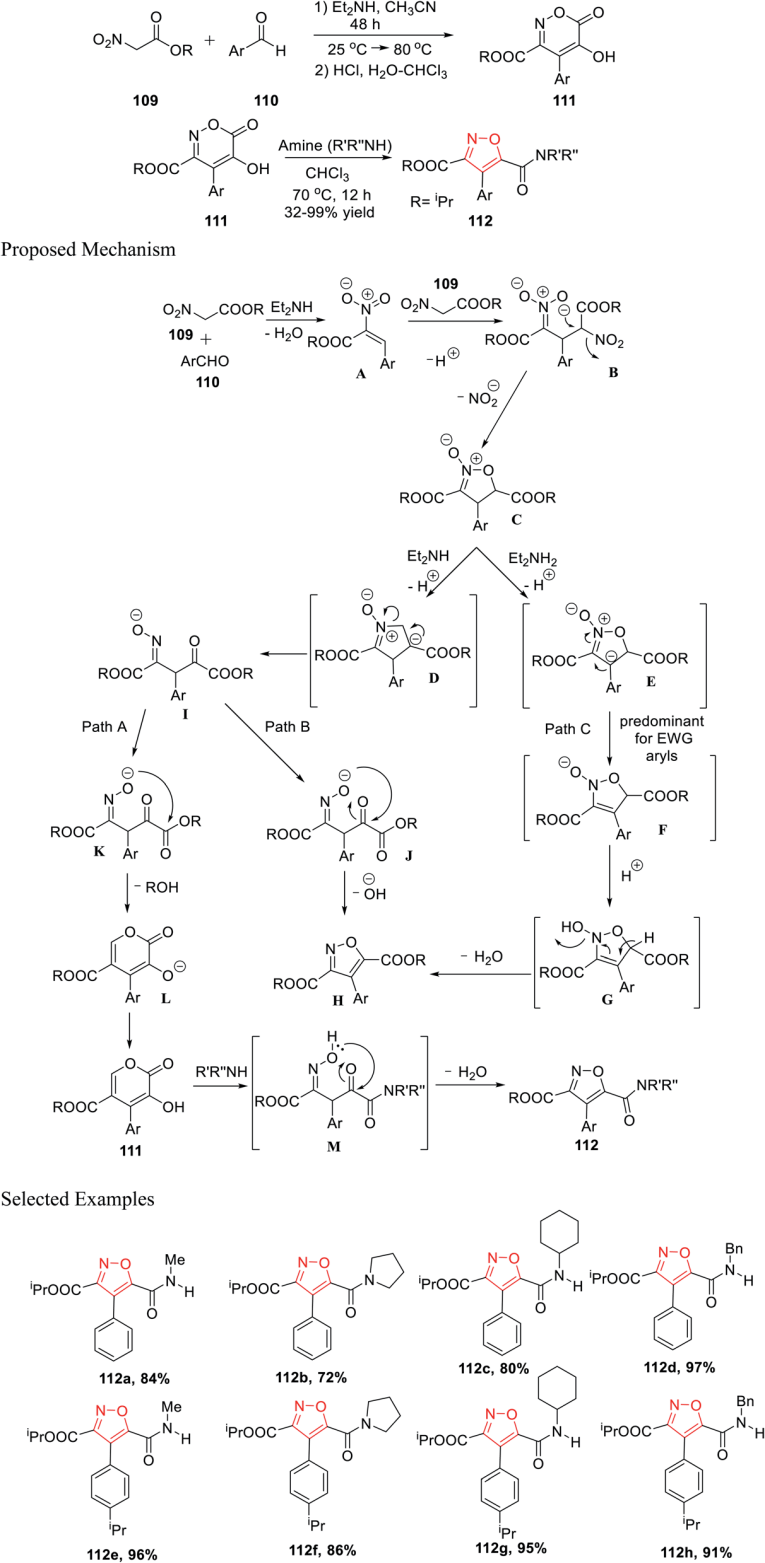
Preparation of 3,4,5-trisubstituted isoxazole derivatives from aromatic aldehyde and nitroacetic esters.

Initially, aromatic aldehydes 110 were reacted with nitroacetic esters 109 in the presence of diethyl amine while using acetonitrile as the solvent for 48 h from 25 °C to 80 °C. Subsequently, the reaction mixture was treated with HCl and hydrated chloroform, and yielded the intermediate alkyl 5-hydroxy-6-oxo-4-aryl-6*H*-1,2-oxazine-3-carboxylate derivatives 111. Subsequently, intermediates 111 were reacted with various functionalized amines in the presence of chloroform at 70 °C for 12 h, which furnished the 3,4,5-trisubstituted isoxazole derivatives 112 (Scheme 27).^39^

Mechanistically, the synthesis of isoxazole began with the formation of the *N*-oxide intermediate C through intermediate B. Isoxazoline C was deprotonated in the presence of Et_2_NH and produced two different types of intermediates E and D, while the formation of E depends on the Ar group. Subsequently, the oxime I underwent cyclization *via* Path A and Path B, and furnished L (six-membered ring) and H (five-membered ring), respectively.

The transformation to species L and H from I is extremely sensitive and depends on the medium properties. Hence, compound 111 was formed *via* protonation from L. Finally, compound 112 was formed from compound 111 through the formation of intermediate M, followed by water elimination. Afterward, substituted isoxazole H was formed by water elimination from E (Path C).

**Scheme 28:**
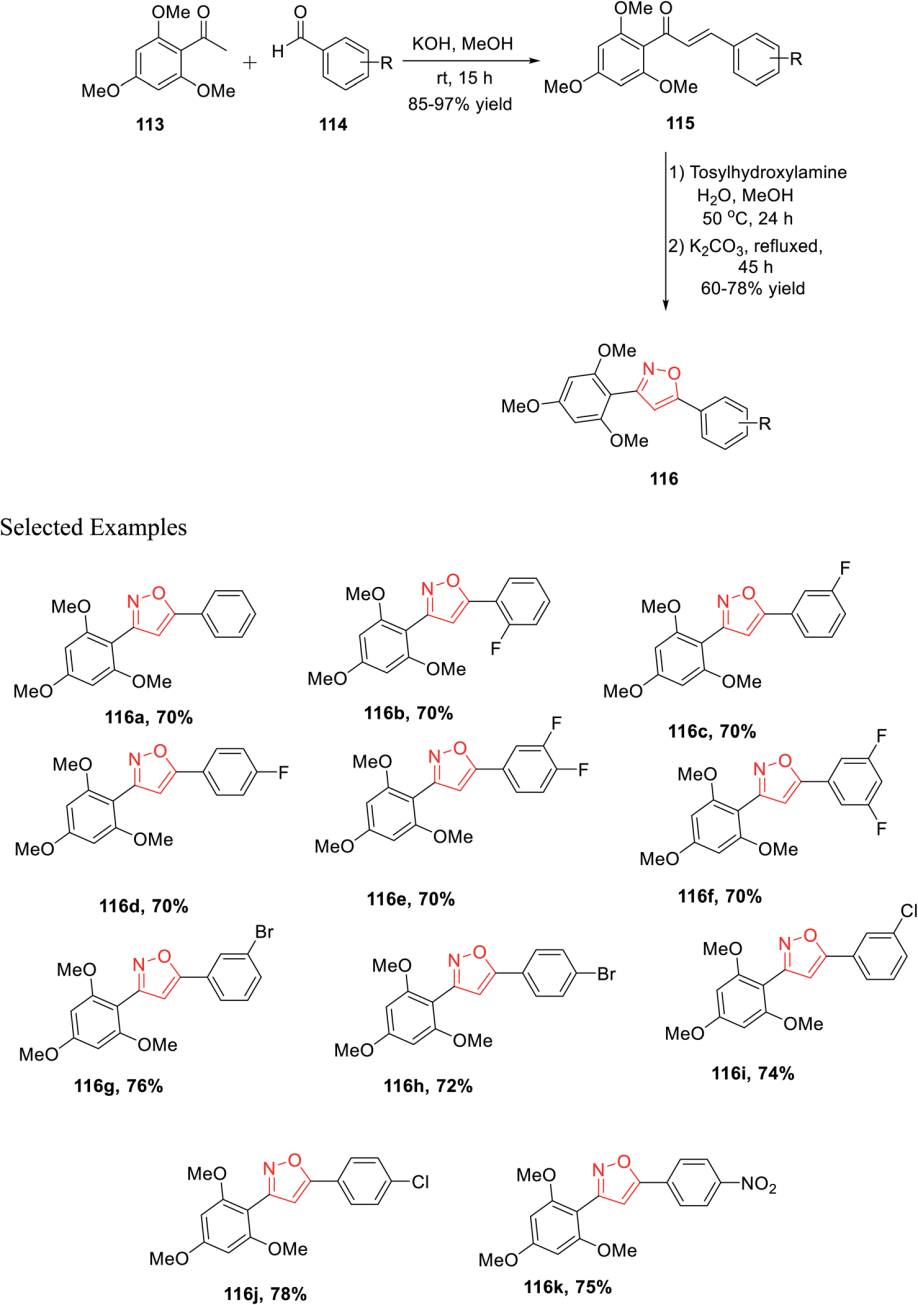
Preparation of 3,5-diaryl isoxazole scaffolds *via* the chalcone intermediate.

In the last year, Burmaoglu and his co-workers demonstrated a synthetic strategy and biological study of 3,5-diaryl isoxazole scaffolds as effective anticancer mediators. Initially, 2,4,6-trimethoxy acetophenone 113 was reacted with functionalized arylaldehydes 114 in the presence of methanolic KOH at room temperature for 15 h, which afforded functionalized chalcone derivatives 115. Subsequently, in the next step, chalcones 115 were reacted with tosylhydroxylamine in aqueous methanol solvent for 24 h at 50 °C and later K_2_CO_3_ was added and refluxed for 45 h, which furnished 3,5-diaryl isoxazole moieties 116 (Scheme 28).

The biological potential (selectivity and activity) was tested *via* cancer PC3 cells and non-tumorigenic PNT1a cells for prostate cancer using all the derivatives 116(a–k). Interestingly, compounds (according to their selectivity in the decreasing order) 116c, 116g, 116d, 116f, 116b, 116k, 116e, and 116a were highly selective as well as active toward the cancer cells, which is comparatively better than that of typical epithelial cells. Inadequately, compounds 116h, 116i, and 116j did not exhibit such selectivity toward the cancer cells. Surprisingly, compound 116c showed the maximum selectivity value, which is comparable with the value of 5-FU. Remarkably, compounds 116a displayed the lowest IC_50_ value with the value of 3.40 ± 0.52 and 2.66 ± 0.22 for PNT1a and PC3, respectively.^40^

Later, Chitneni and his group accomplished the synthetic methodology as well as the antibacterial activity of unique cinnoline-isoxazole hybrid scaffolds. Initially, anthranilic acid derivatives 117 were reacted with methyl lithium in anhydrous THF solvent for 3 h at −78 °C to produce the corresponding *o*-amino acetophenones 118. Afterward, the *o*-amino acetophenones 118 were treated with NaNO_2_, conc. HCl, urea, and sodium acetate in aqueous medium to produce the corresponding cinnolin-4(1*H*)-one derivatives 119 in excellent yields. Subsequently, the cinnolin-4(1*H*)-one derivatives 119 were reacted with propargyl bromide in the presence of K_2_CO_3_ in refluxing acetonitrile medium for 8 h to obtain the crucial intermediate cinnoline scaffolds 120.

**Scheme 29:**
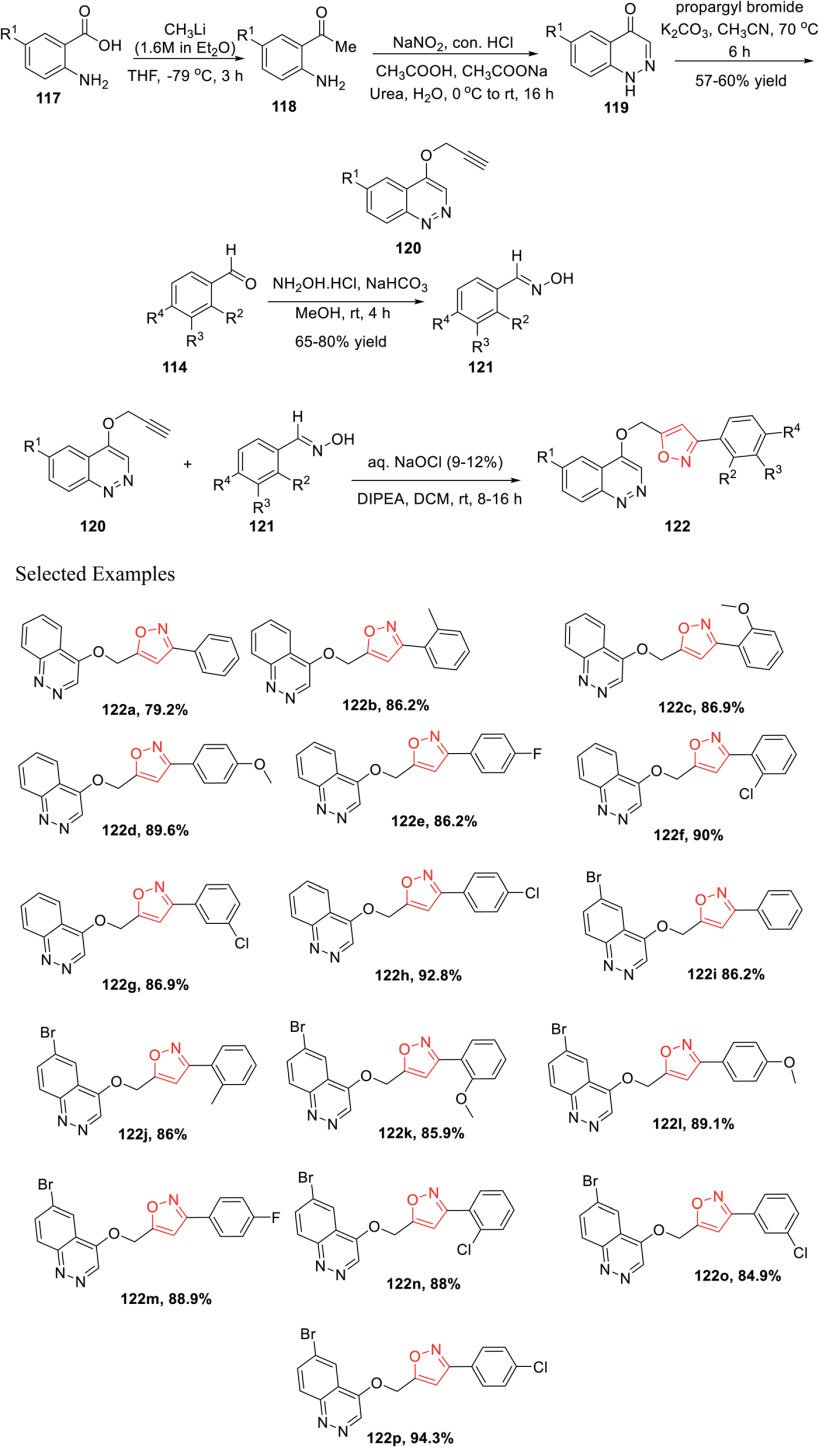
Synthesis of unique cinnoline-isoxazole derivatives.

Again, other main intermediates were synthesized *via* the reaction of functionalized aromatic aldehydes 114 and hydroxylamine hydrochloride in mild basic condition under methanol medium at room temperature for 4 h to provide the corresponding oxime derivatives 121. Finally, cinnoline scaffolds 120 were reacted with the synthesized oxime derivatives 121 in the presence of aqueous sodium hypochlorite and DIPEA in dichloromethane solvent at ambient temperature for 8–16 h, which furnished the corresponding cinnoline-isoxazole scaffolds 122 with an excellent yield of 65–80% (Scheme 29). All the compounds of 122(a–p) were tested for the antibacterial activity toward the human pathogens, Gram-positive bacteria such as *Staphylococcus aureus* and *Bacillus subtilis*, and Gram-negative bacteria such as *Pseudomonas aeruginosa* and *Escherichia coli*. Compounds 122h, 122i, 122j, 122m, 122n, 122o, and 122p displayed good inhibition activity and compounds 122f, 122g, 122k, and 122l displayed excellent inhibition activity toward all the abovementioned bacteria. Interestingly, among all the derivatives, compound 122k showed the best result against *Pseudomonas aeruginosa*.^41^

## Summary

Considering the extensive pharmaceutical usage, isoxazole and its derivatives can be synthesized *via* several synthetic methodologies, which have been established from time to time using microwave, green, solid support, and conventional methods. Herein, we have attempted to explore these methodologies engaged in the formation of isoxazole derivatives due to the easily available low-cost stable starting materials, mild reaction condition, and metal-free reaction condition. The main approach is to synthesize the isoxazole *via* the 1,3-dipolar cycloaddition reaction of dipolarophile and dipole. In this review article, we have attempted to focus on the latest data available on the synthesis and application of isoxazole derivatives from 2010 to 2020 using metal-free synthetic conditions, which include solid support, microwave-assisted, as well as ultrasonication methods. Herein, we have covered the latest and vital biological activities of isoxazole derivatives such as anti-cancer, HDAC inhibitors, antibiotic, COX2 selective inhibitors, neurotoxin, and anti-rheumatic properties. In conclusion, this isoxazole scaffold can play a major role in drug discovery research and these metal-free synthetic methods will encourage the scientific world to engage more and more ‘metal-free syntheses’ in the drug discovery programme.

## Conflicts of interest

There are no conflicts to declare.

## Supplementary Material
